# Detecting copy number status and uncovering subclonal markers in heterogeneous tumor biopsies

**DOI:** 10.1186/1471-2164-12-230

**Published:** 2011-05-11

**Authors:** Fabio Parisi, Stephan Ariyan, Deepak Narayan, Antonella Bacchiocchi, Kathleen Hoyt, Elaine Cheng, Fang Xu, Peining Li, Ruth Halaban, Yuval Kluger

**Affiliations:** 1 Department of Pathology and Yale Cancer Center, Yale University School of Medicine, New Haven, Connecticut, USA; 2 Department of Surgery, Yale University School of Medicine, New Haven, Connecticut, USA; 3 Department of Dermatology, Yale University School of Medicine, New Haven, Connecticut, USA; 4 Department of Genetics, Yale University School of Medicine, New Haven, Connecticut, USA

**Keywords:** copy number, SNP arrays, Next generation sequencing, melanoma

## Abstract

**Background:**

Genomic aberrations can be used to determine cancer diagnosis and prognosis. Clinically relevant novel aberrations can be discovered using high-throughput assays such as Single Nucleotide Polymorphism (SNP) arrays and next-generation sequencing, which typically provide aggregate signals of many cells at once. However, heterogeneity of tumor subclones dramatically complicates the task of detecting aberrations.

**Results:**

The aggregate signal of a population of subclones can be described as a linear system of equations. We employed a measure of allelic imbalance and total amount of DNA to characterize each locus by the copy number status (gain, loss or neither) of the strongest subclonal component. We designed simulated data to compare our measure to existing approaches and we analyzed SNP-arrays from 30 melanoma samples and transcriptome sequencing (RNA-Seq) from one melanoma sample.

We showed that any system describing aggregate subclonal signals is underdetermined, leading to non-unique solutions for the exact copy number profile of subclones. For this reason, our illustrative measure was more robust than existing Hidden Markov Model (HMM) based tools in inferring the aberration status, as indicated by tests on simulated data. This higher robustness contributed in identifying numerous aberrations in several loci of melanoma samples. We validated the heterogeneity and aberration status within single biopsies by fluorescent *in situ *hybridization of four affected and transcriptionally up-regulated genes E2F8, ETV4, EZH2 and FAM84B in 11 melanoma cell lines. Heterogeneity was further demonstrated in the analysis of allelic imbalance changes along single exons from melanoma RNA-Seq.

**Conclusions:**

These studies demonstrate how subclonal heterogeneity, prevalent in tumor samples, is reflected in aggregate signals measured by high-throughput techniques. Our proposed approach yields high robustness in detecting copy number alterations using high-throughput technologies and has the potential to identify specific subclonal markers from next-generation sequencing data.

## Background

The molecular basis for cancer evolution and metastatic spread remain largely unknown. The formidable complexity and evasive nature of tumor biology are the major reasons for the slow progress in implementing effective modalities of prevention and treatment of most cancers. Tumor populations are evolutionarily advantaged because they are dynamic aggregates of constantly evolving subclones, each carrying a variety of genomic aberrations [1]. To date copy number alterations, such as deletion, duplications and amplifications, or spatial genomic re-arrangements, such as translocations and inversions, have been characterized and associated with several different types of cancers [2,3]. In addition, certain sets of aberrations present at diagnosis are expected to be associated with disease recurrence [1]. Identification and characterization of these alterations is of great relevance in understanding the underlying biology of cancer, as well as the design of clinically useful biomarkers of cancer relapse or metastases.

Karyotyping is a standard and effective single cell screening approach that has been used to detect significant genomic aberrations in cancer and in normal populations. Resolution is the main limitation of this technique; the coarse aberration profile obtained by karyotyping is not sensitive enough to detect short, yet relevant, abnormalities [4]. Array-based techniques, such as Comparative Genomic Hybridization (CGH) and Single Nucleotide Polymorphisms (SNP) arrays, have been used as an alternative to conventional cytogenetic approaches in the study of copy number alterations (CNA) in cancer [4]. SNP-arrays generally have a higher resolution than CGH-arrays and can also be used to detect allele-specific information. In addition, genome-wide association studies have employed SNP-arrays successfully to identify copy number variation (CNV) in normal and diseased populations [5-7]. In contrast to traditional karyotyping, SNP-arrays offer the possibility of investigating up to 10^6 ^loci at once for several million cells in a single experiment. However, these high-throughput approaches do not reveal the full genealogical perspective of the tumor biopsy. Instead, they measure aggregate signals of multigenerational progeny. While this is not an issue when analyzing a homogeneous population of cells, it negatively affects the feasibility of correct CNA identification and classification of tumor samples. In contrast to germline samples, aggregate signals of tumor biopsies exhibit a higher degree of complexity due to the extent, variety and frequency of aberrations, contamination from stromal cells and the intrinsic heterogeneity of cancer [8]. Heterogeneity, in particular, reflects the dynamic nature of tumors as aggregates of different subclones, each carrying a continually varying number of genomic aberrations. Progress in effective treatment of early stage cancers will likely be restricted by this immense complexity until we are able to find new methods to separate, catalog and classify the subclones that are responsible for invasion and metastases.

In order to systematically investigate the effects of subclonal heterogeneity in the quantitative analysis of cancer biopsies, we studied the feasibility of uncovering subclonal components and their relative abundance from aggregate signals using linear algebra. Motivated by the need of CNA detection measures that are robust to tumor heterogeneity we defined a class of measures that can be used to classify CNAs from tumor biopsies into three categories: gain, loss and normal. We extended the application of this class of measures to demonstrate how heterogeneity is reflected in data derived from transcriptome massive parallel sequencing (RNA-Seq). Altogether, our study addresses some of the challenges arising in analyses of aggregate signals at the DNA and RNA levels of heterogeneous tumor samples. To our knowledge, the present work is the first to explicitly address the limitations imposed by subclonal heterogeneity in high-throughput experiments and to discuss the tractability of copy number inference from aggregate signals of subclones in mathematical terms.

## Methods

### De-mixing of samples

In a homogeneous sample *s*, the exact genotype of each cell in the sample can be represented by the pair of vectors, A and B, containing respectively the number of copies of the A- and B-allele at each locus. For the sake of simplicity, we assume that each locus is at most bi-allelic, namely that only two variants, A and B, can occur. In the case of tumor biopsies, or studies in which cells from several individuals are pooled together, the measured  and  vectors correspond to the average copy number of the A- and B-alleles at each locus across all cells in the sample. When studying heterogeneous samples, it is very unlikely to find any cell in the sample whose genotype corresponds exactly to the measured profile.

The "de-mixing problem" is the task of recovering the fraction *x_i_*, called the mixing coefficient, and the genotype *g_i _*= {*A_i_, B_i_*} of each i-th homogeneous group of cells in the sample (e.g. the different subclones). This problem corresponds to solving the linear system

(1),

where *x_i _*is an element of the vector X, *j *is the locus index, *a_ji _*and *b_ji _*are elements of the matrices *A *and *B *respectively, and *N_0 _*is the set of all non-negative integers.

### Deconvoluting genotyping data signal from heterogeneous cell population

The de-mixing problem is trivially solved when only two independent components (e.g. from two different individuals) are mixed together, and their copy number, *a_ji _+ b_ji_*, is measured using independent experimental approaches in at least two loci. However, in general, deconvolution of CNA signals from a heterogeneous sample to its homogeneous components is an ill-posed mathematical problem. Linear algebra solution approaches will encounter non-unique solutions corresponding to a non-unique combination of possible discrete vectors representing different subclonal aberration profiles.

Regularization methods (such as L1 minimization for recovery of sparse signals) are often employed to reduce the number of feasible solutions in underdetermined problems. The underlying assumption of applying regularization in this context is that a sample will not generally contain all theoretical subclones, defined by all possible combinations of aberrations. Instead, a few subclones arising during cancer progression will be selected. This assumption is also consistent with the possibility that subclones' viability may be hindered by the lethal synergy of some of their aberrations. Donoho and Tanner reported key results on regularization methods from counting faces of randomly projected polytopes when the projection radically lowers dimension [9]. It is possible to show that even in the simplified problem with *a*_*ji *_∈ {0,1} *b*_*ji *_∈ {0,1}, the required condition for sparse reconstruction,(2)

is not fulfilled for any *s *> 2, where 2^s^-1 is the size of the search space, with *s *equal to the number of loci measured, *k *is the number of components (e.g. observable subclones) and *s*+1 is the maximum number of equations, corresponding to the number *s *of unique measurements (e.g. SNPs), including the additional constraint that the sum of mixing coefficients is 1. Thus, a mixture of 3 or more components, which is typical in tumor biopsies, cannot be uniquely deconvolved solving the linear system, or using sparse reconstruction methods. In the presence of heterogeneous samples no inference can be made of the exact copy number from the aggregate signal of the subclones.

To illustrate these points, we provide a detailed example of the conditions and solution of copy number inference for a mixture of a tumor subclone component with its matched stromal component. In this scenario, de-mixing is elicited by additional constraints on the copy number of the stromal and of the tumor components. The stromal component is assumed to have no CNA, thus its number of copies is constant and equal to 2. The linear system in Eq.1 of the measured aggregate genotypes  at the j-th locus is reduced to:

(3),

where *x *is the mixing coefficient of the stromal component, *a_j1 _*and *b_j1 _*are the A- and B-allele copy number of the tumor, *a_j2 _*is the A-allele copy number of the stromal component and *N_0 _*is the set of all non-negative integers. In addition, we include the equation *a_j1_+b_j1 _= m_j _*in the system in order to restrict the copy number of the tumor component at the j-th locus to an integer *m_j_*. We consider only the loci harboring deletion events with an aggregate copy number smaller than two, where m_j _- the copy number of the tumor component - can take one of two values: one if the deletion in the tumor cells is hemizygous, or zero if it is homozygous. We then solve the system at each locus independently for the two possible values of *m_j _*and determine the two corresponding possible values of *x*. Since there is only one tumor component, there must exist one value of *x *to solve Eq.3 for all loci simultaneously. The mode of all values of *x *across all loci corresponds to this value. The corresponding value of *m_j _*at the j-th locus is then the exact copy number of the tumor component.

### A simple framework for CNA analysis

CNA analysis encompasses the tasks of detecting and classifying copy number aberrations. Current HMM based applications for CNA analysis rely on the assumption that increasing the number of hidden states, thereby increasing the complexity of the underlying model, will eventually result in higher CNA classification accuracy [10-12]. As detailed above, determination of the exact number of copies is an ill-posed problem in the presence of heterogeneous samples. Therefore, we propose to detect substantial deviation from the normal diploid state by classifying the aberrations as gains or losses inferred from the dominant component in the mixture. The goal of CNA analysis then becomes to infer the most recurring aberration that would be observed in a hypothetical, high-resolution karyotyping of the same heterogeneous sample, if it were available.

**Figure 1:**
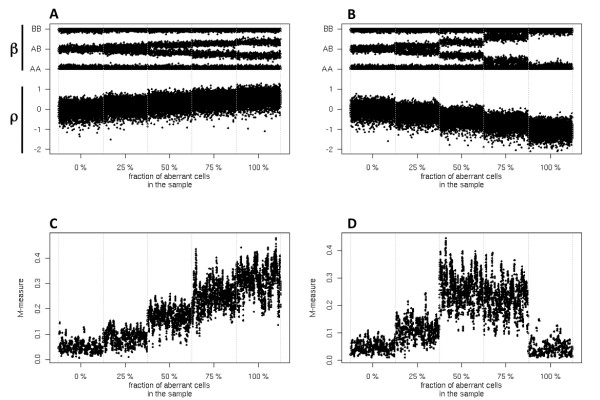
**Simulated binary mixtures of aberrant and normal states**. Mixtures of aberrant and normal states are reflected in deviations from the balance between A and B alleles in heterozygous SNPs and can be detected by simple measures. In panels A and B we display simulations of log R-ratio (ρ) and B allele frequency (β) spanning 1000 loci for mixtures of a normal state with a copy number of 2 with an aberrant state derived from this normal state with a copy number of 3 or 1. As the fraction of aberrant cells increases, the central band of the B-allele frequency, which exhibits the balance between A and B alleles, deviates to the outer band, indicating the dominance of one of the two alleles. The M-measure shown in panels C and D is a detector of allelic imbalance corresponding to the simulations shown in panels A and B, respectively. A limitation of the M-measure is that deviations of the M-measure from 0 for pure deletion states are indistinguishable from deviations in pure stromal states. This can be easily overcome by testing whether the log R-ratio is significantly below its expected value of 0. The combination of the log R-ratio and the M-measure indicates whether the aberrant state is a deletion (log R-ratio < 0) or duplication (log R-ratio > 0).

Peiffer et al. [13] employed a convenient transformation of *A- *and *B-*allele copy numbers to *B-*allele frequency β and the total-DNA enrichment ρ. For clarity of discussion, we use a simpler version of this transformation for each SNP defined as:

(4),

where *N *is the total number of cells in the sample,  are the allele counts estimated from the SNP-arrays. Peiffer and et al. suggest estimating this quantity from the signal of a normal population (e.g. HapMap samples [5]). In general, occurrence of CNA is reflected in the total-DNA enrichment. In the presence of heterogeneous samples, these changes can be difficult to detect due to the high level of noise. As expected, in heterogeneous samples, aberrations also lead to allelic imbalance at positions of heterozygous SNPs (Figure 1). We thus propose to base CNA detection in heterogeneous samples on measures of allelic imbalance.

One such measure of allelic imbalance is

(5),

where W corresponds to a window of appropriate size. This quantity is an aberration score for each SNP, based on the measure of allelic imbalance in its neighborhood. We denote this measure as the M-measure and use it for the remainder of the analyses with W = 20. W = 20 is an arbitrary choice of a window large enough to have a reasonably robust estimate of the M-Measure. The M-measure is insensitive to hemizygous deletion events if they occur in the majority of cells in the sample. We address this issue by testing whether the total-DNA enrichment ρ, also known as R-ratio, is significantly below its expected value of 1. We use the M-measure to classify SNP CNA profiles into three states: gain, loss and neither. The M-measure is a measure of deviation from the expected state of normal non-tumor sample. In order to have a measure that is robust to noise, we chose trigonometric functions such that their product is close to zero in a wide region around the value of the expected state of a normal, non-tumor sample. These trigonometric functions have a non-linear rapid change of the deviation score once the threshold of having a true gain or loss is crossed. As previously discussed, in this context there are numerous solutions to the problem of inferring the exact number of copies consistent with the measured aggregate cell population signal (Eq.1). As none of the possible solutions can be preferred to the others, characterization of CNAs in terms of gain and loss is a practical alternative.

The three-state M-measure classification can also be used in a three-state Viterbi algorithm whose hidden states are normal, deletion, and duplication segments. We name this classification approach 3SMM. We use 3SMM only in our simulated scenarios. In order to use the best parameters for our simulations, we determined transition and emission probabilities from the underlying true copy number status of the simulated data for each run.

### Simulated data

We randomly generated two haplotypes of 10,000 loci to simulate a mixture of a homogeneous tumor subclone and its matched stromal cells. A haplotype is a vector of zeros and ones, representing the two alleles respectively. Our random generation process ensures that at each locus, the germline had a 90.5% probability of being homozygous and a 9.5% probability of being heterozygous. The stromal (non aberrant) component of the mixture was obtained by combining the two haplotypes as described in Eq.1. The tumor (aberrant) component was obtained by adding aberrations to the two haplotypes. To have a unique profile of aberration status, we excluded simultaneous occurrence of different kinds of aberrations (e.g. deletion and duplication) at the same locus. We mixed the normal and aberrant components with proportions of α and (1-α) respectively. Gaussian noise with a signal-to-noise ratio of 30 was used to simulate experimental noise.

We then simulated samples comprising two tumoral subclones and their matched stromal component. As in the case with one subclone, we excluded simultaneous occurrence of different kind of aberrations (e.g. deletion and duplication) at the same locus in the same subclone (e.g. neutral LOH); however, the two tumor components were generated such that their aggregate signal contained all combinations of aberrations (e.g. deletion and duplication). The three components were then mixed by a linear combination with proportions of α, 2(1-α)/3 and (1-α)/3 corresponding to the normal component and the remaining two tumoral subclones respectively. As in the case with two components, Gaussian noise with a signal-to-noise ratio of 30 was used to simulate experimental noise.

We tested four different types of aberrations: homozygous deletion, hemizygous deletion, a gain of one copy, and a gain of two copies. We did not test neutral LOH since it is not an event that would affect the copy number. Each aberration covers 1,000 loci (~20 Mbps). Aberrations were separated by 1,000 aberration-free loci (~20 Mbps). Our aim was to test the classification performance of different approaches to heterogenous-like data rather than testing the sensitivity to detect short focal events. This motivated our choice of aberrations of 1000 loci each.

### Measure of performance

To assess algorithmic performance we used the Area Under the Receiver-Operator Characteristic Curve (AUCROC) measure. In the present study, which is different from the usual formulation, in which the classification task is to distinguish between a set of positive instances and a set of negative instances, we had three class labels: gain, loss and normal. In a typical machine learning scenario in which there is no parameter to vary, the AUCROC in the operative point (which is also termed balanced accuracy) is computed as

(6),

where *TP *is the number of true positives, *P *is the number of positives, *TN *is the number of true negatives and *N *is the number of negatives. Similarly, in our three-classes scenario, the AUCROC can be computed as

(7),

where *TP_gain _*is the number of SNPs that are amplified and correctly inferred as such by the algorithm, *P_gain _*is the total number of SNPs that are amplified in the sample, *TN_gain _*is the number of SNPs that are not amplified and that are correctly inferred as such by the algorithm, *N_gain _*is the total number of SNPs that are not amplified in the sample; *TP_loss_* is the number of SNPs that are deleted and correctly inferred as such by the algorithm, *P_loss _*is the total number of SNPs that are deleted in the sample, *TN_loss _*is the number of SNPs that are not deleted and that are correctly inferred as such by the algorithm, *N_loss _*is the total number of SNPs that are not deleted in the sample; *TP_normal_* is the number of SNPs that are not aberrated and that are correctly inferred as such by the algorithm, *P_normal _*is the total number of SNPs that are not aberrated in the sample, *TN_normal_* is the number of SNPs that are aberrated and correctly inferred as such by the algorithm, and *N_normal _*is the total number of SNPs that are aberrated in the sample.

### SNP profiling using microarrays

DNA from 30 melanoma cell lines were hybridized to Illumina's Human1M BeadChip (Illumina Inc. San Diego, CA). We generated B-allele frequencies and Log-R ratios using standard procedures included in the Illumina BeadStudio package. We normalized with respect to the population of western European ancestry (CEU) from the HapMap project that was analyzed on the Illumina Human1M BeadChip.

### Design, probe annotation, and data processing of the arrays for detection of genome-wide gene expression

We used NimbleGen genome-wide human expression arrays (2005-04-20_Human_60mer_1in2) with a total of < 400,000 probes for < 30,000 transcripts and < 20,000 known genes, as of NimbleGen annotations. NimbleGen provides design and probe annotation. Standard methods for one-channel and two-channel microarrays from the R statistical software were used as previously described [14].

### Transcriptome profiling using next-generation sequencing

We re-analyzed the RNA-seq sample MeWo from a recent study [15,16]. Namely, the reads were aligned to the reference genome using Bowtie [17] with standard parameters. Nucleotide variations were determined after pileup using Samtools [18], and the frequency of the variant, *β*, was calculated as in Eq.4.

### Fluorescent in situ hybridization (FISH)

Fluorescence in situ hybridization (FISH) was performed using probes from the bacterial artificial chromosome (BAC) clones (RPCI-11 human BAC library) containing the selected genes *E2F8 *(248D22 and 80B10 at 11p15.1), *ETV4 *(100E5 and 147C10 at 17q21.31), *EZH2 *(140E16 and 24N19 at 7q36.1) and *FAM84B *(455K11 and 90G11 at 8q24.21). All BAC clones were cultured in 100 ml LB media supplemented with chloramphenicol at 37°C shaker incubator overnight, and cell pellets collected by centrifuge were used for DNA extraction using the large-construct kit (Qiagen, Valencia, CA). Two BAC clones for the 5'-end or the 3'-end of each gene were labeled differently by SpectrumGreen-dUTP or SpectrumOrange-dUTP using the nick translation kit (Abbott Molecular, Des Plaines, IL). Probe hybridization on slides of interphase cells was performed following the laboratory's standardized protocol. Hybridization signals were visualized and captured using an Olympus BX60 fluorescence microscope with CytoVision software version 4.5.2 (Genetix, San Jose, CA). In each sample, 200 nuclei were inspected and the signal patterns were documented.

## Results

### The measure of allelic imbalance (M-measure) is robust to heterogeneity

We performed simulations to study the behavior of the allelic imbalance M-measure in presence of aberrations (Figure 1C and Figure 1D). A simple threshold procedure can be used to identify high confidence CNA loci. An arbitrary choice of 0.1 for the cutoff of the M-measure and window size W = 20 is sufficient to achieve satisfactory accuracy (Figure 1). Remarkably, the M-measure is robust in detecting aberrations even when the aberrated component is present at low concentrations (Figure 1).

Comparison of the performance of the M-measure to that of state-of-the-art HMM-based CNA methods requires data in which the subclonal composition and copy number of each subclone are known. Currently, there is no practical solution to catalog all aberrations in all clones in a given sample, and we therefore used simulated data to test the accuracy of CNA classification of complex mixtures. Following the binary mixture procedure described in the Methods section, we generated 200 independent datasets for selected values of the mixing coefficient α to simulate a scenario of contaminating a homogeneous tumor sample (composed of only one subclone) with stromal cells. This binary mixture scenario should not be confused with the one used by Peiffer et al [13]. Peiffer et al. mixed tumor samples of one individual with normal cells from another individual, generating a mixed sample that was not reflective of clinical settings. In our simulations, we mixed components from the same individual, reflecting stromal contamination, which is often present in tumor samples. Deconvolution strategies to analyze mixtures comprising one normal and one homogenous tumor component have been proposed by others [10,13].

**Figure 2:**
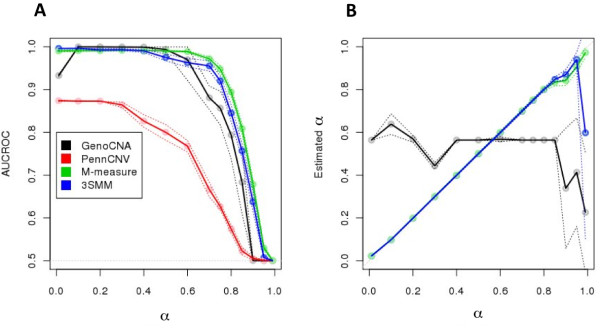
**Comparisons between performance of the M-measure, HMM-based CNA algorithms and the three-state Viterbi decoding based on the M-measure (3SMM) for binary mixtures**. We simulated 200 datasets reflecting the mixture of a homogeneous tumor sample and a stromal component at different levels of the mixing coefficient. Panel A shows the average of the areas under the ROC curve (AUCROC) at the operative point for the correct classification of the three states (Eq.7) of GenoCNA (black), PennCNV (red), the M-measure (green) and a three-state Viterbi decoding based on the M-measure, 3SMM (blue). The dotted envelope around each curve represents two units of standard deviation centered at the mean performance. Overall, the M-measure performs equally well or better than the HMM-based algorithm. B. Inference of the mixing coefficient according to the genoCNA algorithm (black) or to the solution of the linear systems (Eq.3) in regions classified as loss based on the M-measure classification (green) or 3SMM (blue). The dotted envelope around each curve represents two units of standard deviation around the mean of the inferred coefficient. The value of the mixing coefficient, which is inferred from the classification using the M-measure and/or 3SMM, is not surprising as it reflects the deterministic nature of the underlying linear system. On the other hand, the low performance of genoCNA is attributed to the non-uniqueness of solutions in the problem of inferring exact copy numbers, particularly amplifications.

We tested PennCNV [11], genoCNA [10], the M-measure and the three-state Viterbi algorithm based on the M-measure (3SMM, see Methods) using the mixture scenario of a stromal contamination with a "pure" tumor sample. We used default parameters for both PennCNV and genoCNA. As a measure of performance, we calculated the average of the areas under the receiver-operator characteristic curve (AUCROC) at the operative points (Eq. 7), reducing the classification task to the correct classification of gain, loss and normal states (Figure 2B). As expected, PennCNV performed the worst. PennCNV is not designed for tumor copy number inference. Its poor performance indicates that algorithms that do not take into account the possibility that the data could be a mixture of more than one component will perform worse than algorithms that do not ignore this possibility. Given the low performance, PennCNV was not included in any further analysis. Overall, the M-measure performed as well as, or better than, the state-of-the-art HMM-based methods. Remarkably, it exhibited higher robustness to the mixing coefficient. Interestingly, 3SMM shows no evident improvement compared to the M-measure (Figure 2A, blue and green AUCROCs). We also compared the actual value of the mixing coefficient to the value inferred by solving the linear system, as described in the Methods, and to the value returned by genoCNA, as described in the genoCNA documentation. As shown in Figure 2B, our solution obtained using linear algebra is close to the actual solution (green and blue curves), even when the normal component was overwhelmingly abundant. The main effect of increasing stromal contamination seems to be reflected in a slightly larger variance of the estimated mixing coefficient, which is particularly visible in the inference based upon the 3SMM algorithm. This increase in the variance reflects the overall decrease in performance in terms of AUCROC (Figure 2A green and blue curves). The surprisingly weak performance of genoCNA in inferring the correct mixing coefficient is probably linked to the non-uniqueness of solutions in the problem of inferring exact copy numbers. As the use of Viterbi decoding did not significantly improve algorithmic performance, we did not use 3SMM for further analyses.

**Figure 3:**
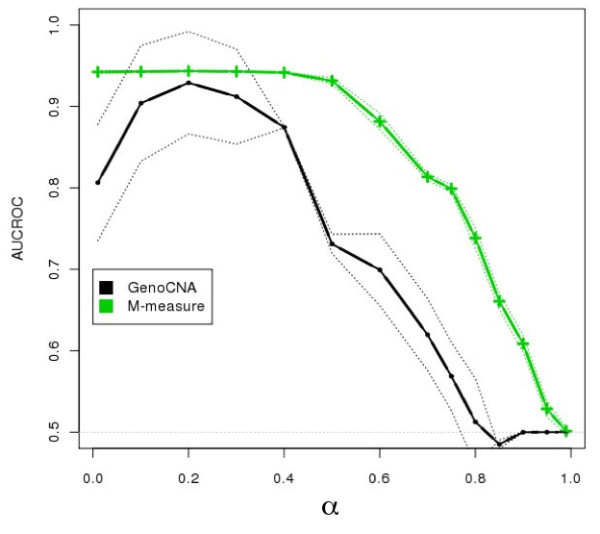
**Performance comparisons between HMM-based CNA algorithms and M-measure for mixtures of three states**. We simulated 200 datasets by mixing a stromal component with relative concentration of α, with two independent tumor components with relative concentrations of 2(1- α)/3 and (1- α)/3, respectively. To assess performance, the output of these algorithms is first classified into three states (gain, loss and normal), and the average between the AUCROCs at the operative points (Eq.7) is computed for GenoCNA (black) and the M-measure (green). The correct state is defined as the state of the aberrant signal with the largest coefficient. The dotted envelope around each curve represents two units of standard deviation, and the central solid line represents the mean performance. Overall, the M-measure (green) exhibits a high degree of robustness to increasing levels of stromal contamination. Real samples will generally have more than two components, including the stromal fraction. Therefore, although genoCNA is not designed to handle heterogeneous samples with more than two components, its performance has been added to the plot to show the way in which state-of-the-art HMM-based methods perform.

To test classification performance in more heterogeneous conditions, we generated 200 independent datasets of a tumor sample with two subclones contaminated by a stromal component for selected values of the normal component mixing coefficient α, as described above. In this scenario, the M-measure clearly shows its superior robustness in the inference of CNA status from heterogeneous samples with an AUCROC above 0.9 for mixtures in which the tumor components together constitute at least half of the sample (Figure 3). The classification performance of the M-measure decreases as the total fraction of stromal cells increased, suggesting that the decreasing signal-to-noise ratio of allelic imbalance, as already shown in Figure 1, is the main cause for misclassification. Further comparison using real SNP-array tumor data revealed higher robustness of the M-Measure in identifying de novo aberrations during tumor progression (Additional file 1, Additional file 1, Figure.S1, Additional file 1, Figure.S2 and Additional file 1, Table S1).

In general, the linear relationship between probe signal and allelic content shown in Equation 1 recapitulates that pooling and counting the number of occurrences of each allele from a biological sample follows basic algebraic relationships. This is the case in karyotyping, FISH experiments, exome sequencing, DNA-seq and many other experimental procedures. Equation 1 does not refer to a specific technology; instead, it reflects basic statistics notions and is, in its theoretical formulation, exact. It has been shown that SNP-array data may require preprocessing steps involving non-linear transformations [19]. We have shown that copy numbers cannot be inferred at the linear level and that *a fortiori *cannot be inferred after applying the non-linear transformations required to faithfully analyze SNP-array data.

### Experimental validation of robustness of the allelic imbalance measure to heterogeneity

**Figure 4:**
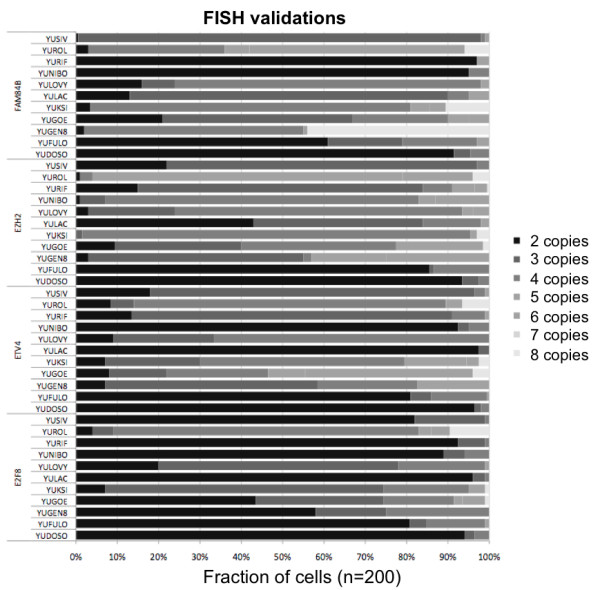
**Fluorescent in situ hybridization (FISH) analysis of four candidate genes that are up-regulated in melanoma and have a high M-measure**. Two probes were used for each gene: one probe covers the 3'-end and the other probe covers the 5'-end. For each gene in each sample, 200 cells were analyzed, and the frequency of aberrations is reported. No translocation events were detected. The spectrum of aberrations of the selected four genes is highly heterogeneous, such that each cell has a different number of amplifications (black and grey bars). With the exception of YUDOSO, at least one locus among FAM84B, ETV4 and EZH2 are aberrant in more than 75% of the cells in the tumor samples.

We sought to demonstrate that the allelic imbalance measure can detect CNA in highly heterogeneous tumor samples. For this purpose, we selected loci from melanoma tumor samples (Table 1 for details on the samples) that exhibited deviations relative to normal melanocytes in terms of CNA and mRNA expression. We identified 299 genes that were over-expressed in more than 24 melanoma samples (> 80% of the 30 samples in the cohort) relative to normal melanocytes. We selected the 20 most frequently over-expressed genes for further analyses by intersecting these genes with all aberrant genes whose M-measure and log-R were indicative of gain in copy number. Fluorescent in situ hybridization (FISH) analysis of four genes, E2F8, ETV4, EZH2 and FAM84B, which show the most recurring gains, revealed remarkably complex mixtures with varying gains and number of subclones (Figure 4). The prevalence of copy number gains of ETV4 and FAM84B suggests that aberrations involving these genes may be functionally involved in maintaining the disease, or that they occurred early in tumor genesis and inherited by most subclones during tumor expansion. This finding is consistent with melanoma cytogenetic reports of recurring early aberrations of their hosting chromosomes [3].

**Table 1:** Characterization of samples by disease status, stage and BRAF or NRAS mutation

Sample_ID	Normal/Nevus/Melanoma	Stage	BRAF status	NRAS status
HFSC	Normal	Normal	NA	NA
Nbmel	Normal	Normal	NA	NA
YULOVY	Melanoma	I, primary	WT	Q61L
YUPLA	Melanoma	II	WT	WT
YUGOE	Melanoma	III	WT	WT
YUKIM	Melanoma	III	WT	Q61R
YUROL	Melanoma	III	WT	WT
YUPAO	Melanoma	III, acral	WT	WT
YUCAS	Melanoma	IV	WT	WT
YUCHER	Melanoma	IV	WT	Q61R
YUMAG	Melanoma	IV	WT	Q61R / WT
YUROB	Melanoma	IV	WT	WT
YUSIV	Melanoma	IV	WT	WT
YUTUR	Melanoma	IV	WT	WT
YUZOR	Melanoma	IV	WT	WT
YUWERA	Melanoma	IV, acral	WT	WT
YUHOIN	Melanoma	IV, primary	WT	WT
YUDOSO	Melanoma	llb, primary	WT	Q61K / WT
YUHEIK	Melanoma	primary	WT	WT
YUFULO	Melanoma	primary	WT	Q61L / WT
YUSTE	Melanoma	III	V600E	WT
YUCAL	Melanoma	IV	V600E	WT
YUSAC	Melanoma	IV	V600E	WT
YUGEN8	Melanoma	IV	V600E	WT
YUCLIR	Giant nevus	Giant nevus	V600E / WT	WT
YUSIK	Melanoma	III+	V600E / WT	WT
YUNIBO	Melanoma	IIb, primary	V600K	WT
YUKSI	Melanoma	IV	V600K	WT
YULAC	Melanoma	IV	V600K	WT
YUMAC	Melanoma	IV	V600K	WT
YURIF	Melanoma	IV	V600K	WT
YUSIT	Melanoma	IV	V600K / WT	WT

Samples have been characterized by disease status, stage and BRAF or NRAS mutation.

### High-resolution profiling of heterogeneity using next-generation sequencing

**Figure 5:**
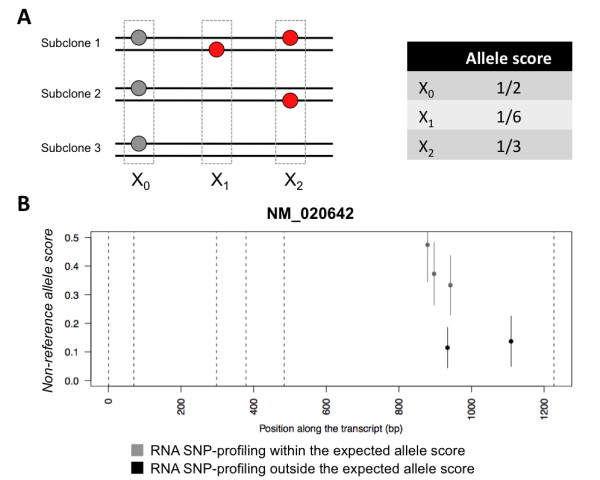
**Inference of subclonal heterogeneity from RNA-Seq data**. The scheme shown in panel A demonstrates the way in which allelic imbalance along a single exon arises. The exon of interest (black lines) carries a germline polymorphism on one of the copies in position X_0 _(grey dot). In addition, out of three sub clones, one acquired a somatic point mutation in position X1, and two acquired somatic point mutations in position X2 (red dots). Assuming that both alleles are expressed with equal frequencies and that the expression level is the same across all subclones, the measured allelic imbalance is determined by the unequal distribution of somatic mutations, as shown in the table. Differences of allele-specific expression levels or of the subclonal expression levels affect the exon as a whole, but do not change the overall picture of distinct allelic imbalances at the X_0_, X_1 _and X_2 _loci. Allelic imbalance along AKIP1 (NM_020642) shown in panel B reflects subclonal heterogeneity. The score of the non-reference nucleotide has been calculated as 0.5 - | 0.5 - (#B/(#B+#A)) |, where #A is the number of reads matching the reference sequence, and #B is the number of reads with a mismatch at the given nucleotide (non-reference nucleotide). The AKIP1 exhibits multi-modal B-allele frequency at heterogeneous nucleotides. Vertical bars represent 95% confidence interval of the estimated proportion, assuming an underlying binomial distribution. All non-reference nucleotides shown are supported by at least 100 reads. Vertical dashed lines mark the boundaries between exons in the transcript.

Allelic imbalance can also be used to detect heterogeneity using next-generation sequencing, in particular genomic DNA sequencing, mRNA sequencing (RNA-seq) and whole-exome sequencing. Similar to the CNA analysis, allelic imbalance at the single nucleotide level can give insights into tumor heterogeneity, provided that there is sufficient sequencing coverage to reliably identify variations distinctive of subclonal populations. A recent study produced an in-depth sequencing profile of melanoma samples [15,16]. We re-analyzed sequencing data from the MeWo melanoma cell line, which had the highest sequencing depth, to identify patterns associated with subclonal heterogeneity. Inferring heterogeneity from RNA-Seq is very difficult due to the co-occurrence of numerous effects, such as allele-specific expression, varying expression levels across subclones, and alternative splicing, all of which can alter the allelic balance. To overcome the presence of confounding effects, we focused on the variation of single nucleotide allelic imbalance along exons. We argue that in the absence of subclonal heterogeneity and of CNA at the level of DNA, allelic imbalance is expected to be constant along the entire exon. On the other hand, in the presence of subclonal heterogeneity, acquired variations in some subclones will introduce allelic imbalance at a population level with significant fluctuations between neighboring loci (Figure 5A). This imbalance is not associated with differential expression between paternal and maternal alleles, but rather with the co-occurrence of subclonal variants whose observed expression level may reflect their relative abundance. In addition, large fluctuations between contiguous nucleotides can exclude the presence of focal CNA, which would instead result in highly correlated fluctuations at each nucleotide along the aberration. Given the lack of independent CNA information for the MeWo sample, we studied heterogeneity in RNA-seq using single-nucleotide mutations. However, the proposed approach is general enough to be extended to the study of CNA heterogeneity in next-generation sequencing.

As proof of concept, we examined all nucleotide variants occurring along any exon. To achieve statistical significance, we discarded all nucleotide variants that were supported by less than 100 reads in each sample. We identified several genes in different samples that had signs of significant allelic imbalance at the RNA level. In particular, we observed significant fluctuations in allelic imbalance at different consecutive positions within the same exon that were not associated with indels (Table 2). RNA-editing and short CNAs could be considered an alternative explanation for the observed imbalance. One approach to exclude the occurrence of RNA-editing would be to sequence the genomic DNA of these exons. However, we note that the majority of the reported examples did not correspond to the typical C→U and A→I RNA-editing modifications. In principle, the presence of very short CNAs can be excluded, verifying the signal obtained on high-resolution SNP-arrays. Notably, in some cases, the distance between loci with significantly different allelic imbalance is shorter than the RNA-seq read length; the presence of a CNA common to all subclones would simultaneously affect both loci, leading to similar allelic imbalances. Taken together, these results suggest that these base variations affect only a fraction of the subclones in the sample (Figure 5B and Additional file 2). In conclusion, allelic imbalance measures, besides being robust to heterogeneity in high-throughput analyses, can also be used to detect heterogeneity and uncover specific subclonal markers from next-generation sequencing.

**Table 2:** Subclonal point mutations

Refseq gene	position in transcript	Reference	Variant
NM_001077619	2874	A	G/C
NM_001113202	1035	G	A
NM_001113202	1038	T	A
NM_001113202	1034	T	A
NM_003112	4016	G	A
NM_003112	4024	T	A
NM_003112	4042	C	T
NM_005431	1294	C	G
NM_005431	1325	T	C
NM_005431	1408	A	G
NM_013276	2909	A	G
NM_013276	3065	C	T
NM_013276	3110	G	T
NM_018129	1977	C	T
NM_018129	2044	C	T
NM_018129	2154	A	G
NM_018129	1973	C	A
NM_018129	2822	G	C
NM_018373	1930	G	A
NM_018373	1887	C	T
NM_020642	934	C	T
NM_020642	942	T	C
NM_020675	1099	G	T
NM_020675	1129	G	T
NM_031886	3289	A	G
NM_031886	3285	C	T
NM_031886	3278	G	A
NM_031886	3271	G	A
NM_033426	3002	C	T
NM_033426	2821	A	C
NM_033426	2856	C	T
NM_144578	3320	C	A
NM_144578	3317	T	C
NM_144578	3425	C	T
NM_144578	3948	G	C
NM_144578	3946	G	A
NM_145280	1865	G	A
NM_145280	1623	G	A
NM_145280	1866	C	T/G
NM_145280	3564	A	G
NM_145280	2096	T	C

The majority of these mutations are not indels or typical RNA-editing substitutions, supporting the idea that these point mutations occur in a fraction of the subclones from the heterogeneous melanoma sample.

## Discussion

High-throughput technologies can effectively replace cytogenetics to generate high-resolution maps of chromosomal aberrations. Cataloging potential markers at different length-scales, such as whole chromosome deletions, to few genes, or even to specific nucleotide mutations, has enabled the association of important biological mechanisms with tumor formation and progression. However, the caveat of interpreting data generated by these techniques is that signals measured from tumor biopsies can be an aggregate profile of different cells. To better understand the potential of high-throughput technologies, our study addresses two issues: i) the effects of subclonal heterogeneity on CNA analysis; ii) the identification of copy number alteration measures that are robust to heterogeneity.

In our work we show that heterogeneity has a hindering effect on CNA analyses. Currently there is no direct mathematical procedure to correctly infer the copy number of a heterogeneous sample when the number of homogeneous components is greater than two. Even in toy-models, in which the main focus is on the aberration status rather than on the actual copy number (binary encoding in vectors of 0 and 1's), the search space of aberration profiles grows exponentially with the number of measurements.

On the other hand, at this point, it is important to note that most aberrations span several loci; thus, measurements from SNP-arrays, or from next-generation sequencing techniques will be grouped in clusters of statistically indistinguishable numerical values. As a result, the number of unique measurements is generally smaller than the total number of SNPs on an array, or than the number of base pairs sequenced using next-generation sequencing techniques. The search space of aberration profiles, however, is too large to lead to a unique solution, even using powerful regularization methods. To address this issue, one has to impose additional mathematical constraints motivated by the specific properties of the biological system under investigation.

Currently, state-of-the-art CNA detectors are model-based, i.e. they attempt to predict the exact copy number status and the genotype by fitting the measured quantities with pre-coded models [10,11,20]. The underlying models have been designed to improve sensitivity. However, when HMM-based approaches are employed in simulated scenarios representing optimal signals, the underlying aberrations are not properly inferred (Figure 2A and Figure 3). We propose to reduce the CNA analysis to discrimination between three distinct states, gain, loss, and normal, to detect the presence and type of the state of the strongest aberrant component in the aggregate signal.

We present a simple and biologically motivated framework to design measures of CNAs based on alteration of total DNA, and allelic balance at heterozygous loci. These measures can be easily implemented for three-state classification tasks using thresholding. Our M-measure is one example selected from a family of measures, and it clearly showed unparalleled robustness to sample heterogeneity, thus leading to improved performance in detecting the presence of CNAs. Interestingly, the three-state Viterbi algorithm based on the M-measure did not show a significant improvement in terms of performance over the M-measure alone. This reflects the fact that the decay in performance as the mixing coefficient of the stromal component increases is mostly due to the decreasing strength of the aberrant signal in the mixture, relative to the constant level of experimental noise. Clearly, the simpler goal of three-state classification based on measures such as the M-measure is easier to meet; the advantage is that this type of measure is also more robust when put to test.

Our proposed framework for detecting CNAs in high-throughput SNP-profiling is a conceptual generalization of a class of empirical measures used to identify CNAs [21] and sample heterogeneity [22] employing massive parallel sequencing of genomic DNA. Here, we seek to demonstrate that they can be used to analyze signals in other types of sequencing experiments such as RNA-seq, exome sequencing or ChIP-seq. High-throughput mRNA profiling of tumors has shown dependency between the copy number status and the expression level of the mRNA product [23]. Measuring allelic imbalance from RNA-Seq experiments is partially associated with copy number status of the underlying genomic DNA, yet, as mentioned above, it is not feasible to correctly infer the exact CNA status due to a variety of confounding effects, including heterogeneity. We therefore do not expect RNA-seq alone to be useful in inferring focal copy number aberrations in cancer samples. Next-generation sequencing signals, however, are suitable for studying heterogeneity and characterizing subclonal components by their collection of specific markers. Subclonal heterogeneity is not reflected in CNA exclusively; it is also reflected in other somatic mutations (e.g. point mutations) and other traits, such as DNA methylation and histone modification.

We analyzed RNA-Seq experiments to unravel subclonal heterogeneity. We show that some of the measured allele-specific expression patterns result from differences in the abundance of subclonal populations, each harboring different acquired mutations (Figure 5A). The reported loci are remarkable examples of novel candidate somatic polymorphisms, likely associated with subclonal populations. This approach has striking conceptual and methodological simplicity, and in the near future deviations in the distribution of allelic imbalances along exons might be used to infer the extent of the sample's heterogeneity. The possibility of identifying novel candidate somatic mutations associated with subclonal populations requires experimental strategies that will enable separating different subpopulations for further analyses.

Our results uncover a large degree of heterogeneity at the level of genomic DNA, both in terms of CNA and point mutations. Interestingly enough, heterogeneity is present in both primary and metastatic tumors, suggesting that the variety of underlying mutations may already be overwhelming at diagnosis. The identification of driver mutations, which typically requires examination of DNA at high resolution, is linked to our ability to detect subclones capable of escaping the selective survival pressure and metastasizing. Detecting potentially rare subclones at this resolution requires very deep sequencing of large cohorts of patients.

## Conclusions

Subclonal characterization of cancer samples is crucial to understanding disease progression (seeds of metastasis) and to studying the presence or emergence of resistant subclones in metastatic cells after drug exposure [1,24]. To have a clinical impact, such characterization should not only identify markers for specific subclones in the sample, as we have shown above, but to estimate the relative subclonal abundance as well. Some remarkable attempts have been made in the direction of characterizing subclones from heterogeneous tumor samples [25,26]. These pioneering efforts are promising and important undertakings addressing the difficult problem of subclonal de-mixing. However, due to the non-unique number of solutions of the underlying linear system (Eq.1), reported results have to be interpreted cautiously, more as a suggestive and intuitive representation of one possible evolutionary summary of the sample, rather than the true representation of the subclonal composition of the sample. While we recognize the potential relevance of novel markers reported in these studies [25,26], neglecting the effect of CNA may lead to incorrect inference of the order and the co-occurrence of genomic aberrations.

Exact assessment of copy number in heterogeneous samples using high-throughput or next-generation sequencing technologies is an ill-posed task. However, characterizing CNA in terms of states of deletion or amplification is still feasible. To achieve this goal, we provide a measure that can robustly characterize the dominant aberrations present in heterogeneous tumor samples. As described above, similar measures to the one we use to characterize heterogeneity in the CNA analysis can be applied to data obtained in next-generation sequencing. Understanding the effect of subclonal heterogeneity on measurements and analysis of tumor biopsies is a necessary step to understanding etiology and evolutionary dynamics of tumors. The ability to decipher subclonal composition from tumor biopsies will likely have a major impact on the development of personalized medicine.

## Authors' contributions

FP and YK designed the study. FP and YK performed the bioinformatics analysis. SA and DN collected the tumor biopsies. KH and EC performed the SNP-array experiments. FX and PL performed the FISH validation analysis. AB and RH supported genomic analysis. FP and YK wrote the manuscript. All authors have read and approved the final manuscript.

## Supplementary Material

Additional file 1 **Detecting copy number status and uncovering subclonal markers in heterogeneous tumor biopsies**. Comparing the M-Measure-based approach to a state-of-the-art CNA algorithm by testing their ability to identify de novo aberrations in SNP-array signals from an evolving tumor at two time points. Click here for file

Additional file 2 **Evidence of subclonal heterogeneity from RNA-Seq data across a collection of melanoma samples**. Allelic imbalance along each gene demonstrates subclonal heterogeneity. The score of the non-reference nucleotide has been calculated as 0.5- | 0.5 - (#B/(#B+#A)) |, where #A and #B are the numbers of reads hosting the reference and non-reference nucleotide respectively. However, all the reported genes have a multimodal distribution of B-allele frequencies along at least one exon. The B-allele frequencies close to 0.5 have been marked in grey, B-alleles that significantly deviate from this cluster are considered acquired somatic mutations (black) and can be explained by subclonal heterogeneity. Vertical bars represent 95% confidence intervals. All non-reference nucleotides shown are supported by at least 100 reads. Vertical dashed lines mark the boundaries between exons in the transcript. Click here for file
