# Supplementary material for: Detecting copy number status and uncovering subclonal markers in heterogeneous tumor biopsies
Source: BMC Genomics. 2011 May 11;12:230. doi: 10.1186/1471-2164-12-230 (PMC3114747; doi:10.1186/1471-2164-12-230)
Supplement: Additional file 1 — Detecting copy number status and uncovering subclonal markers in heterogeneous tumor biopsies. Comparing the M-Measure-based approach to a state-of-the-art CNA algorithm by testing their ability to identify de novo aberrations in SNP-array signals from an evolving tumor at two time points. [file 1471-2164-12-230-S1.DOC]

**Detecting copy number status and uncovering subclonal markers in heterogeneous tumor biopsies**

Fabio Parisi, Stephan Ariyan, Deepak Narayan, Antonella Bacchiocchi, Kathleen Hoyt, Elaine Cheng, Fang Xu, Peining Li, Ruth Halaban and Yuval Kluger

***Comparison of SNP-array signals of a tumor cell-line analyzed at different passage time points***

*To further compare the M-Measure-based approach to a state-of-the-art CNA algorithm, we tested their ability to identify de novo aberrations in an evolving tumor at two time points. For this purpose, we used the YUCAS cell-line. The YUCAS cell-line from the Yale cohort was analyzed at different culture passage times, passage 3 and passage 4, using two Illumina SNP-arrays (see Methods). Comparison of the two signals from these arrays showed changes during culture time in certain genomic locations (Fig.S1). We estimated the measured invariance between the two signals as the fraction of SNPs, whose difference in B-Allele frequencies (BAFs) between the two arrays was smaller than 0.05; or, as an alternative, the fraction of SNPs whose difference in Log-R ratios was smaller than 0.5. The different cutoffs reflect the size of the noise in the BAF and in the Log-R ratio, as can be seen in Fig.S2. We calculated the measured invariance for each autosome independently. We found that the two arrays had a high measured invariance across chromosomes (Table S1).*

*The measured invariance is a rough proxy for underlying changes in the aberration state between passages; however, the measured invariance for the Log-R ratio is affected by noise levels that have the same order of magnitude of the differences between the signals from the two arrays. On the other hand, the measured invariance in the BAF is affected by homozygous SNPs that exhibit no change, leading to a high lower bound of the BAF measured invariance. Altogether, this explains why the ranges of values of the measured invariances do not span the full range between 0 and 1. Despite this, the relative order of the measured invariance across chromosomes is still informative and indicative of the underlying number of SNPs affected by copy number changes between the two passages. For instance, chromosome 2 harbors a chromosome-wide aberration (Fig.S1), which correctly corresponds to the smallest values for the two measured invariances (Table S1).*

*We then applied the M-Measure and genoCNA algorithms to the signals from each of the two arrays independently, as described in the Methods. These two algorithms assign to each SNP an expected aberration status (M-Measure, see Methods) or an expected copy number (genoCNA). For each method, we determined the inferred invariance between the two arrays as the fraction of SNPs, whose inferred class remained unchanged between arrays. We calculated the inferred invariance for each chromosome independently. The inferred invariance varied from 20% to almost 100% for both algorithms (Table S1).*

*We did not perform direct numerical comparisons between the measured and the inferred invariances due to expected differences in the range of values. Alternatively, we performed a correlation analysis between measured and inferred invariances for the autosomes (Table S1). We found that there was a very high level of correlation. The highest correlation was detected between the BAF measured invariance and the M-Measure inferred invariance (0.68, Pearson correlation, p-value=0.0005), while the BAF-measured invariance had a lower correlation with the genoCNA inferred invariance (0.41, Pearson correlation, p-value=0.06). The stronger correlation between the BAF-measured invariance and the M-Measure-inferred invariance reflects the robustness of the M-Measure to complex mixtures (see Results, also Fig.3).*

*We sought to investigate the origin of this difference in robustness by visually inspecting two chromosomes showing large differences in inferred invariance between the M-Measure and genoCNA. From this analysis, we found that the M-Measure shows a slightly higher False Negative rate than genoCNA does (Fig.S1). This is due to our conservative threshold used in the M-Measure (see Methods). On the other hand, it seems that the difference in the correlation is due to the higher False Positive rate of genoCNA (Fig.S1). The higher False Positive rate is a direct result of the larger number of possible copy number states in genoCNA, as opposed to the three aberration states of our framework (gain, loss and neither). In addition, since exact copy number inference leads to non-unique solutions when more than two component are mixed together (see Methods), and because the underlying number of copies in a tumor sample is probably not corresponding to a single number (Fig.4), assigning an exact copy number to each SNP is equivalent to choosing one possible solution at random, thus increasing the chance of disagreement between replicated experiments.*

Table S1: Measured and inferred invariance on autosomes between SNP-arrays of the same cell line (YUCAS) measured at different culture passage time-points

*Description*: Comparison of SNP-array signals of a tumor cell-line analyzed at different passage time points. The M-Measure method and genoCNA were used to compare a tumor cell-line analyzed on SNP-arrays at two different passages.

|  | Measured invariance  (%) | | Inferred invariance  (%) | |
| --- | --- | --- | --- | --- |
|  | Log-R | BAF | M-Measure | genoCNA |
| chr1 | 80.5 | 85.8 | 88.9 | 92.4 |
| chr2 | 75.4 | 70.0 | 20.4 | 36.8 |
| chr3 | 81.4 | 81.6 | 96.3 | 83.5 |
| chr4 | 78.5 | 73.7 | 81.9 | 60.7 |
| chr5 | 81.0 | 73.3 | 49.0 | 41.3 |
| chr6 | 80.3 | 84.3 | 77.6 | 42.0 |
| chr7 | 82.5 | 80.8 | 64.5 | 88.3 |
| chr8 | 79.3 | 83.9 | 71.6 | 62.7 |
| chr9 | 79.2 | 81.0 | 95.5 | 90.1 |
| chr10 | 78.7 | 89.2 | 83.6 | 99.9 |
| chr11 | 80.3 | 86.7 | 88.6 | 95.3 |
| chr12 | 81.5 | 77.2 | 60.8 | 56.8 |
| chr13 | 78.2 | 73.0 | 76.2 | 98.6 |
| chr14 | 80.5 | 80.4 | 70.6 | 66.2 |
| chr15 | 79.9 | 92.2 | 90.2 | 95.5 |
| chr16 | 80.3 | 77.9 | 49.9 | 51.8 |
| chr17 | 80.7 | 84.8 | 88.4 | 84.0 |
| chr18 | 80.5 | 74.8 | 53.5 | 47.8 |
| chr19 | 81.6 | 80.1 | 95.9 | 76.0 |
| chr20 | 81.8 | 82.3 | 73.8 | 83.4 |
| chr21 | 77.4 | 92.1 | 94.4 | 46.0 |
| chr22 | 81.6 | 79.1 | 64.1 | 88.8 |


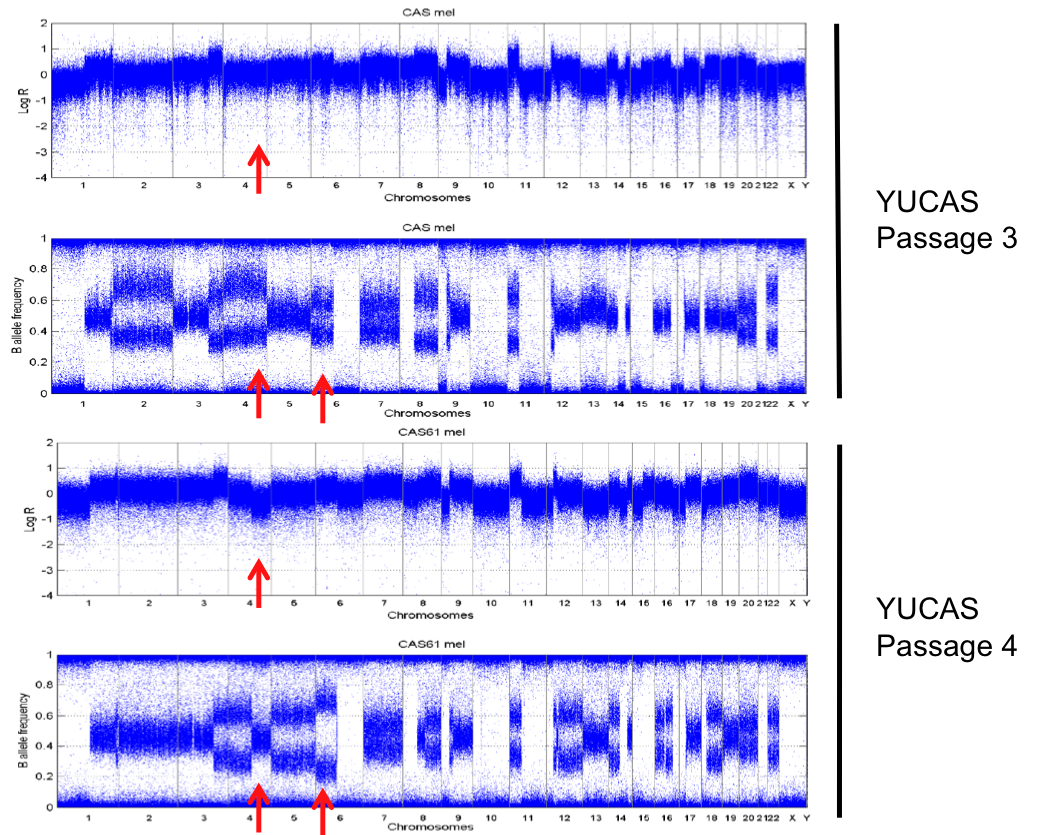


Additional file 1, Figure S1

*Title*: SNP-array profile of YUCAS cell-line at two consecutive early passages.

*Description*: The Log-R ratio and B-Allele frequency genome-wide profiles of the two YUCAS cell-line passages are shown. Black vertical lines indicate the chromosome boundaries. Red arrows indicate illustrative examples of genomic regions with aberration status changes are indicated.


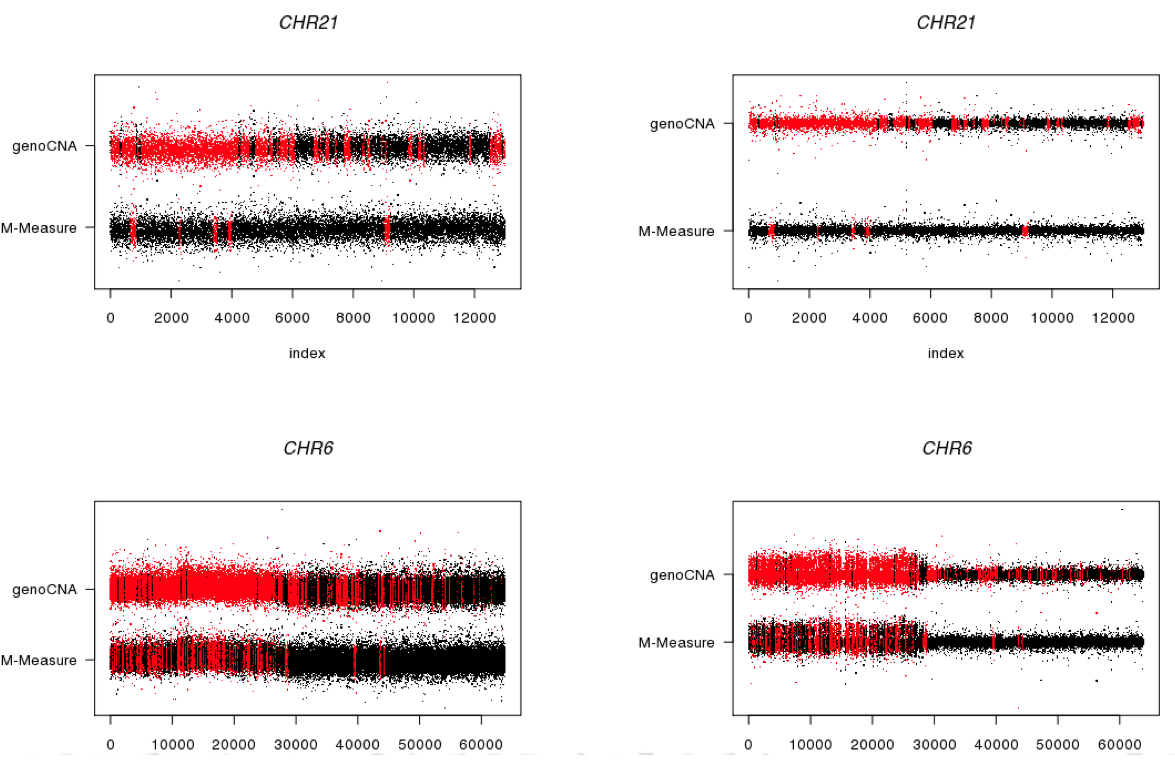


Additional file 1, Figure S2

*Title*: Inferred invariance between SNP-array profiles of two consecutive cell-line passages.

*Description*: The profile of the difference between passage 3 and passage 4 of YUCAS cell-line is shown twice in each plot. Each difference profile is centered on the relative y-axis tick-mark (no difference). Colors correspond to whether the algorithm predictions were invariant (black) or non-invariant (red) between the two signals. No evident change occurs on chromosome 21, as largely inferred by the M-Measure, in contrast to the prediction of genoCNA. On the contrary, a change occurs on chromosome 6, correctly inferred by both algorithms. This change is more clearly reported by genoCNA, which also infers changes in the second half of the chromosome where no visible change takes place.
