# Supplementary figures and images for: Detecting copy number status and uncovering subclonal markers in heterogeneous tumor biopsies
Source: BMC Genomics. 2011 May 11;12:230. doi: 10.1186/1471-2164-12-230 (PMC3114747; doi:10.1186/1471-2164-12-230)

# NM\_001077619

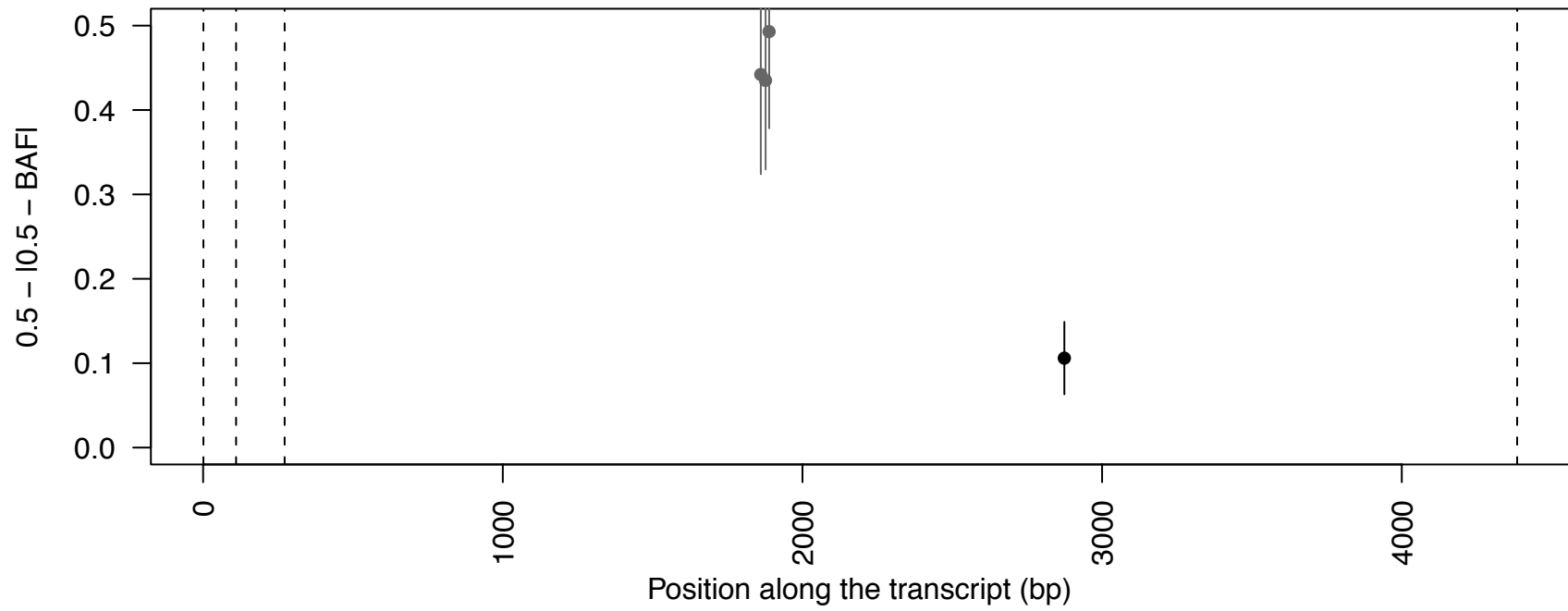

# NM\_001113202

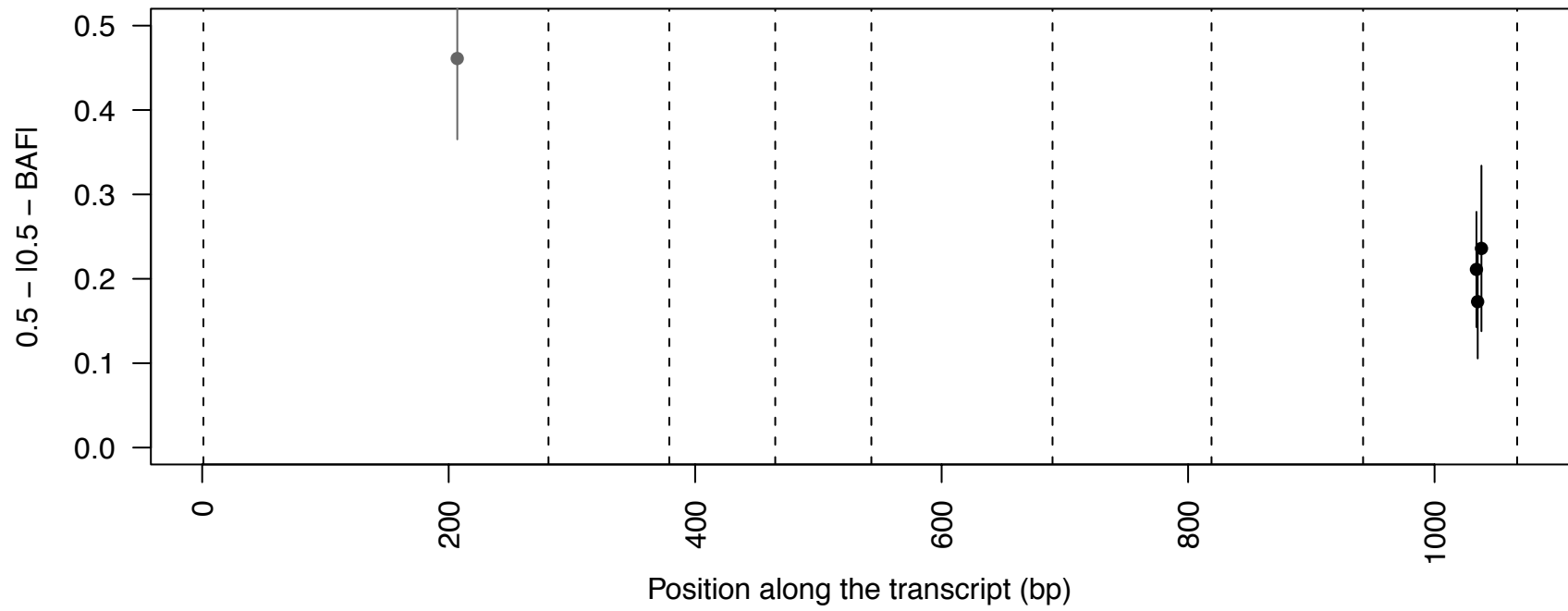

NM\_003112

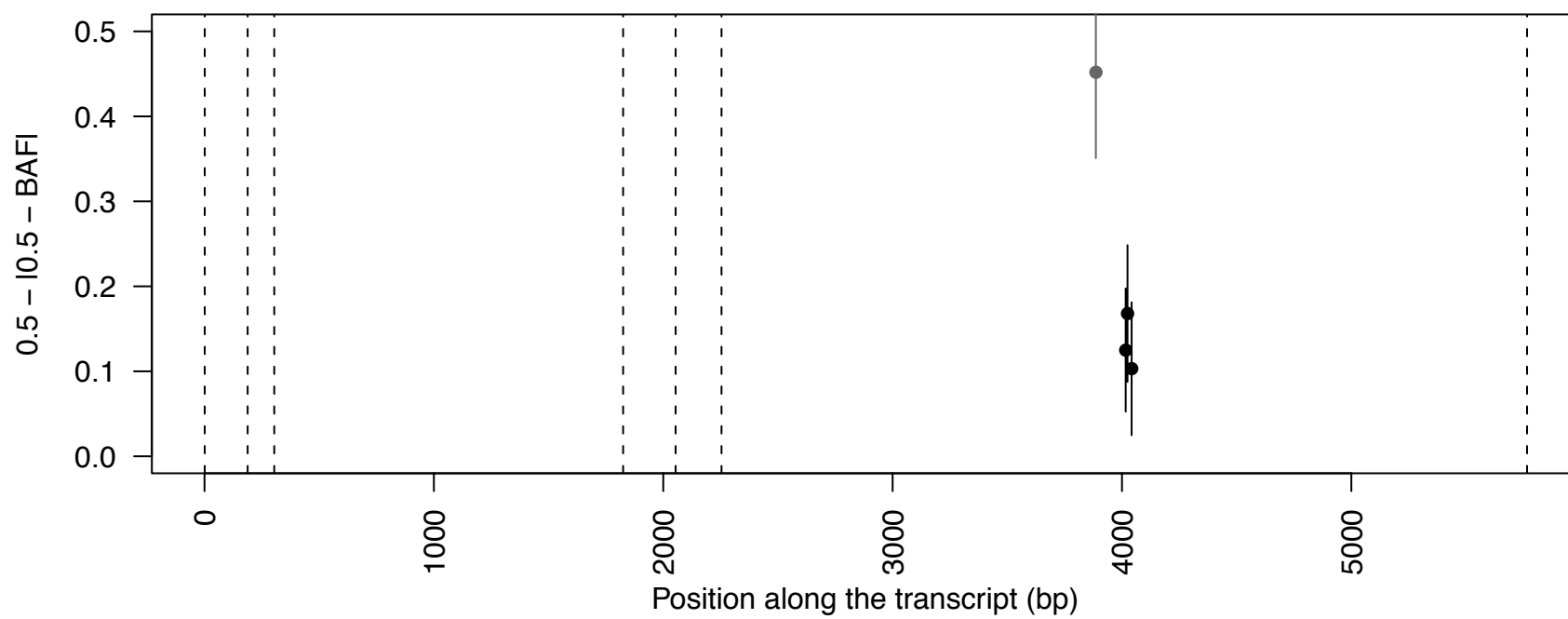

NM\_005431

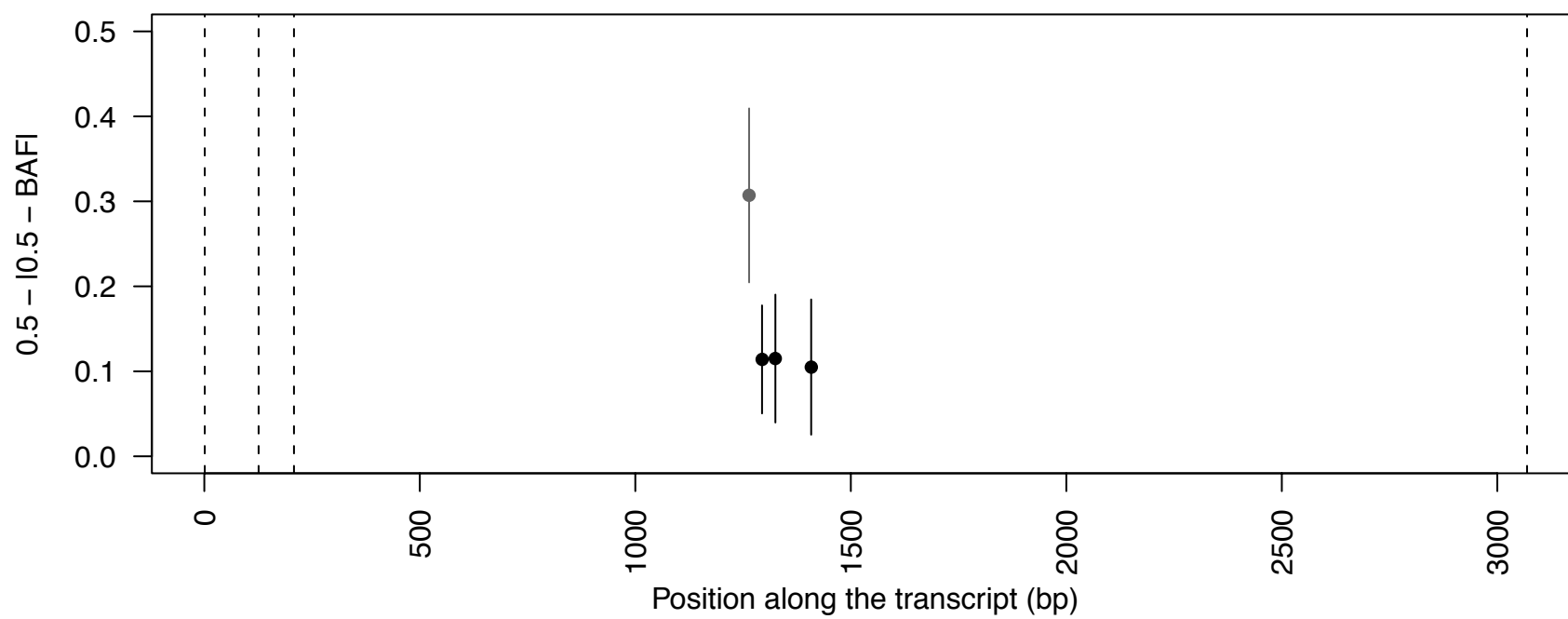

NM\_013276

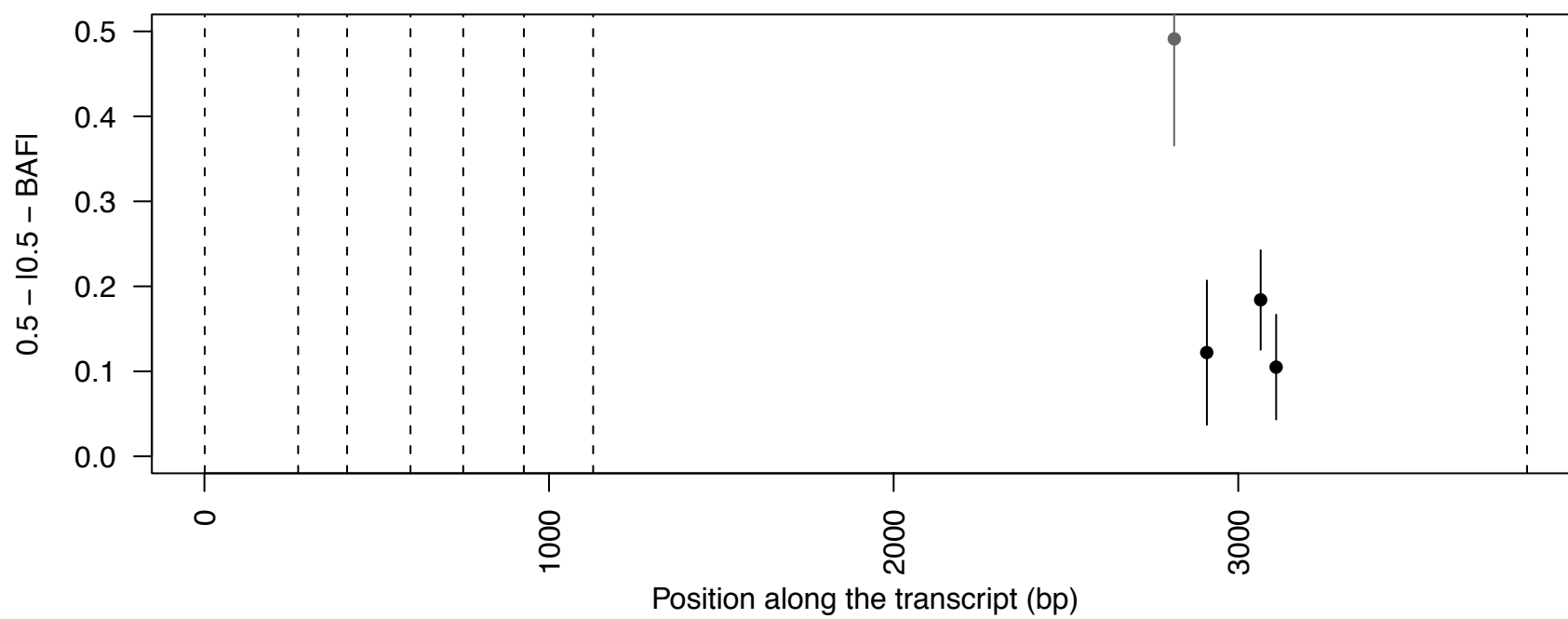

# NM\_018129

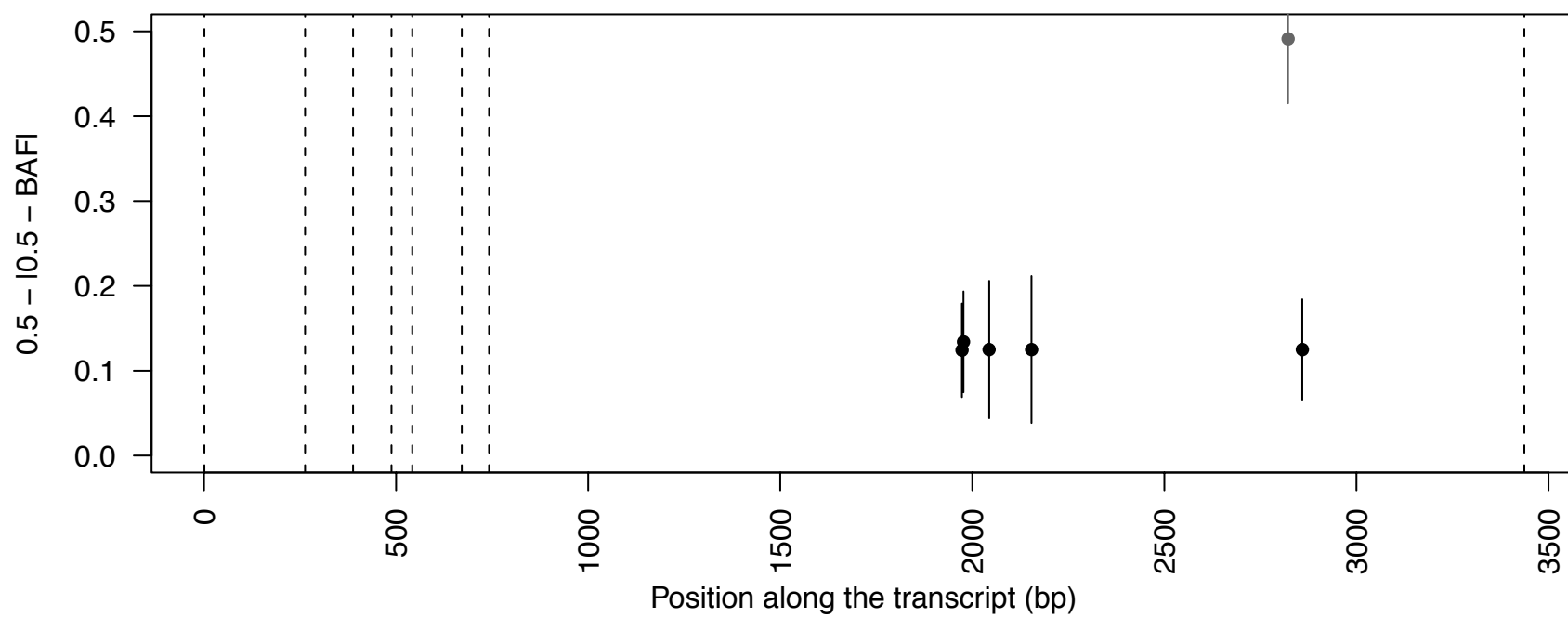

# NM\_018373

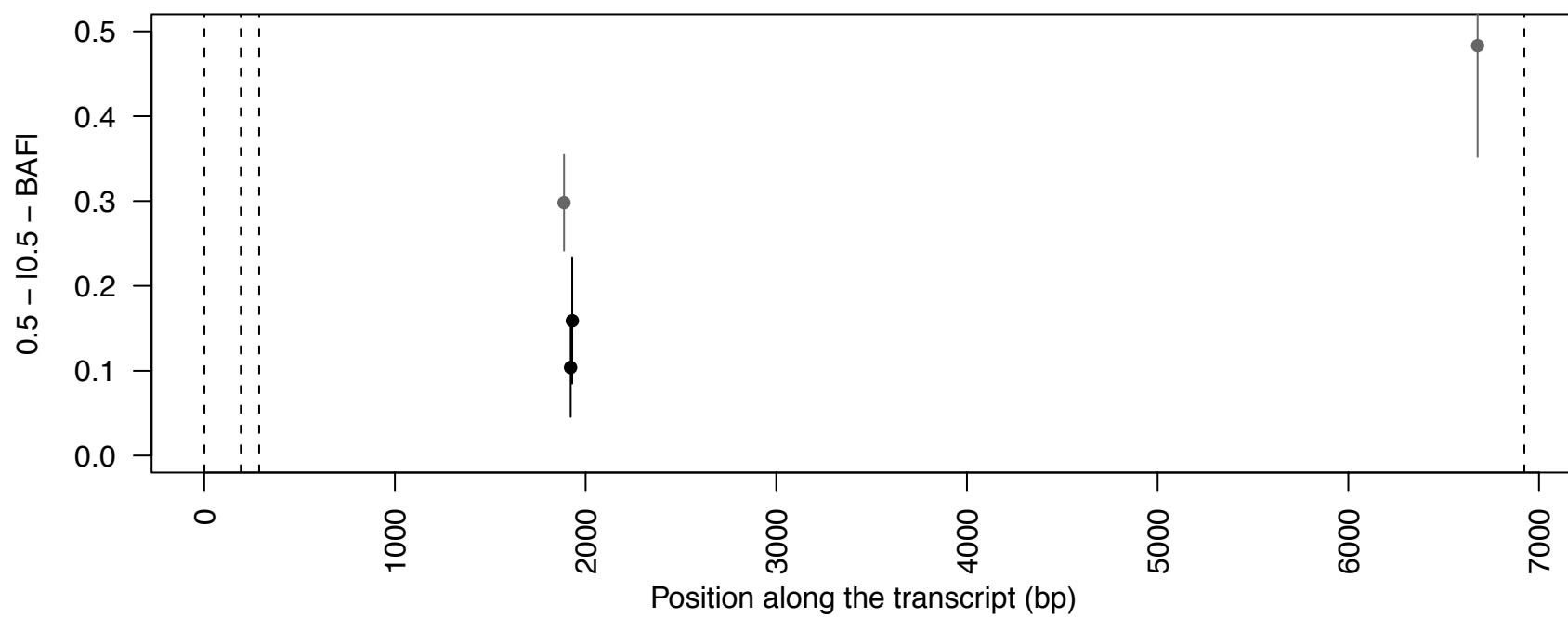

NM\_020642

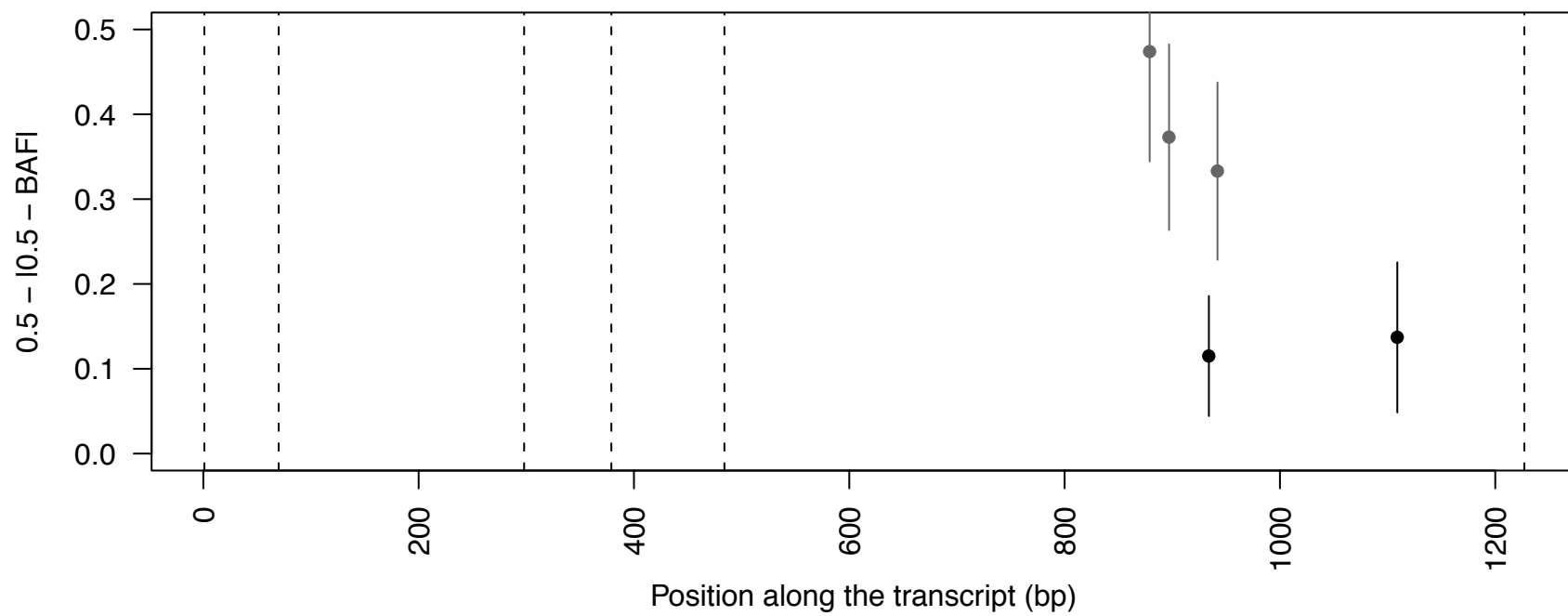

NM\_020675

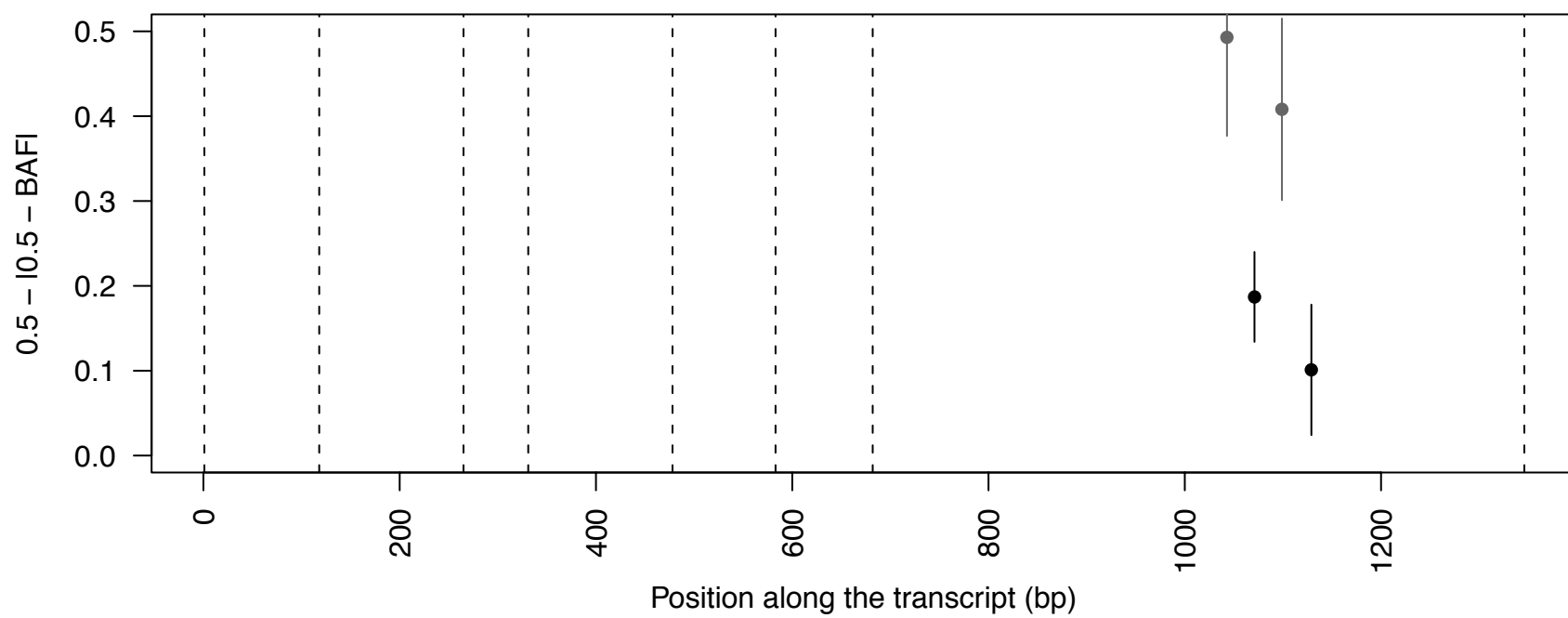

# NM\_031886

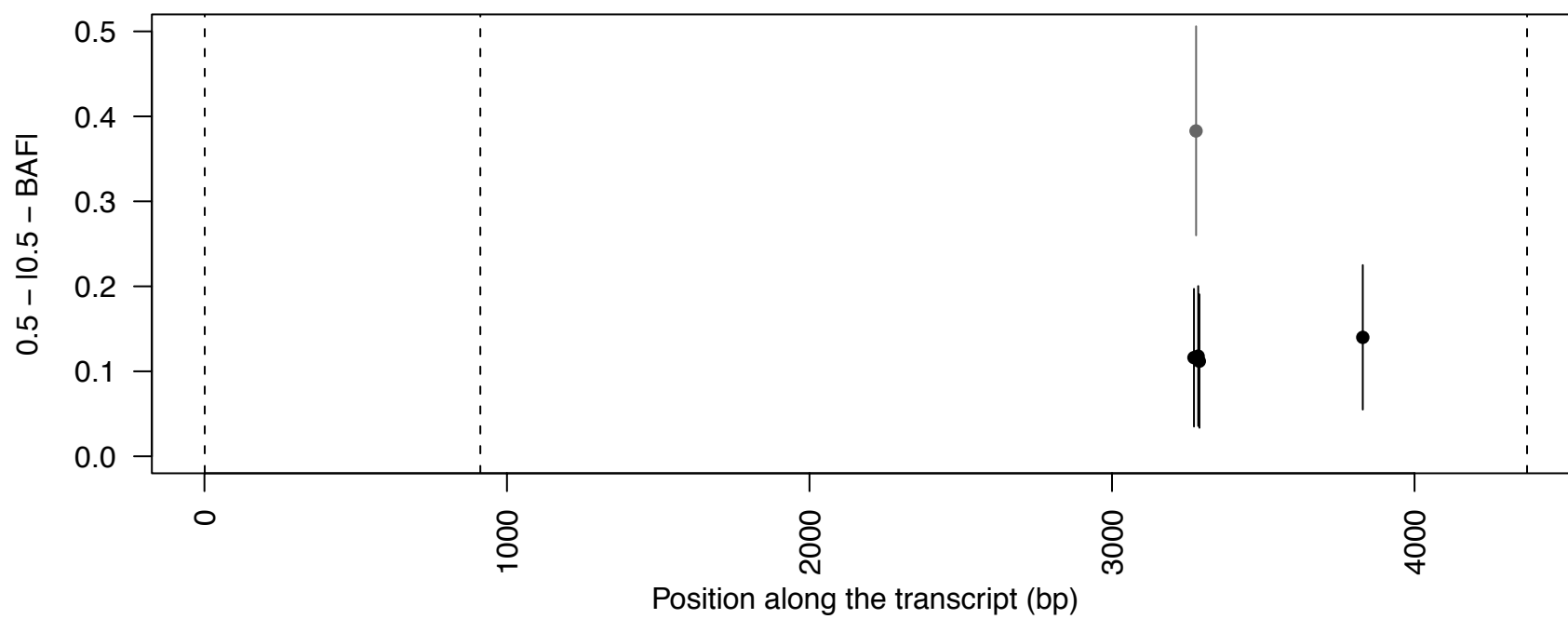

NM\_033426

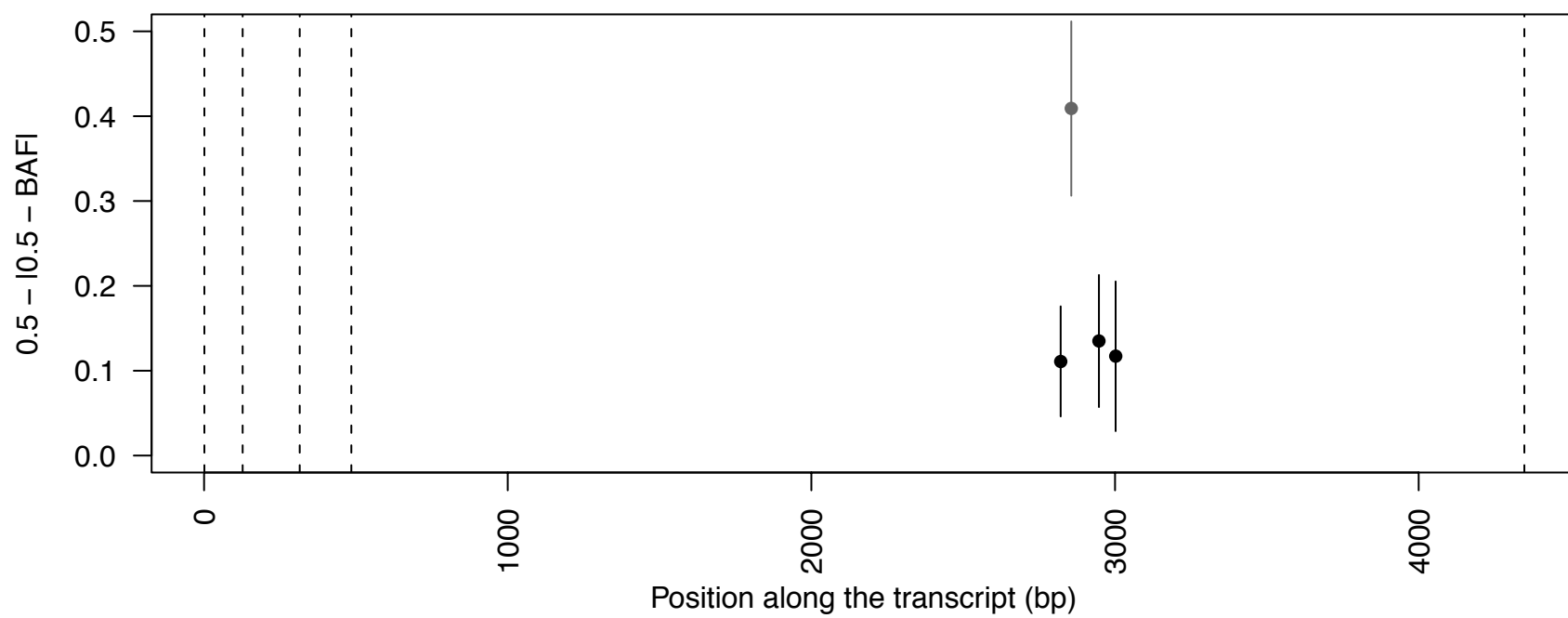

NM\_144578

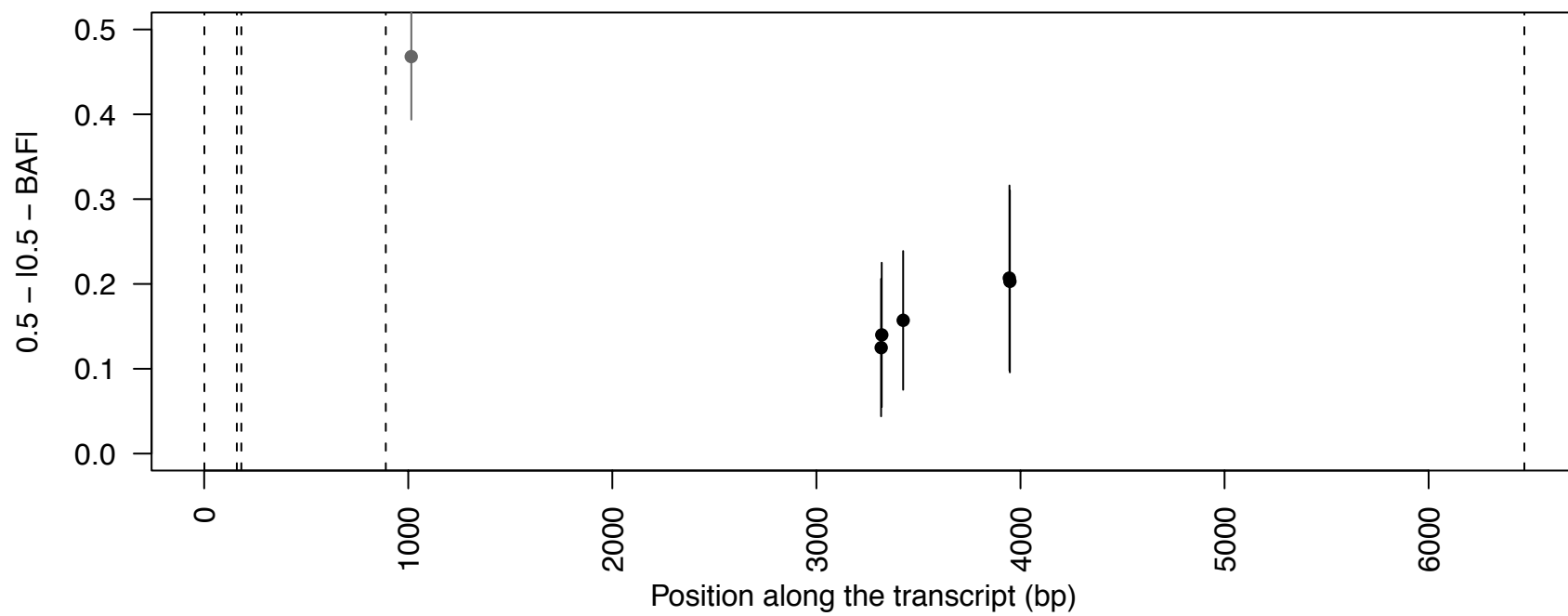

# NM\_145280

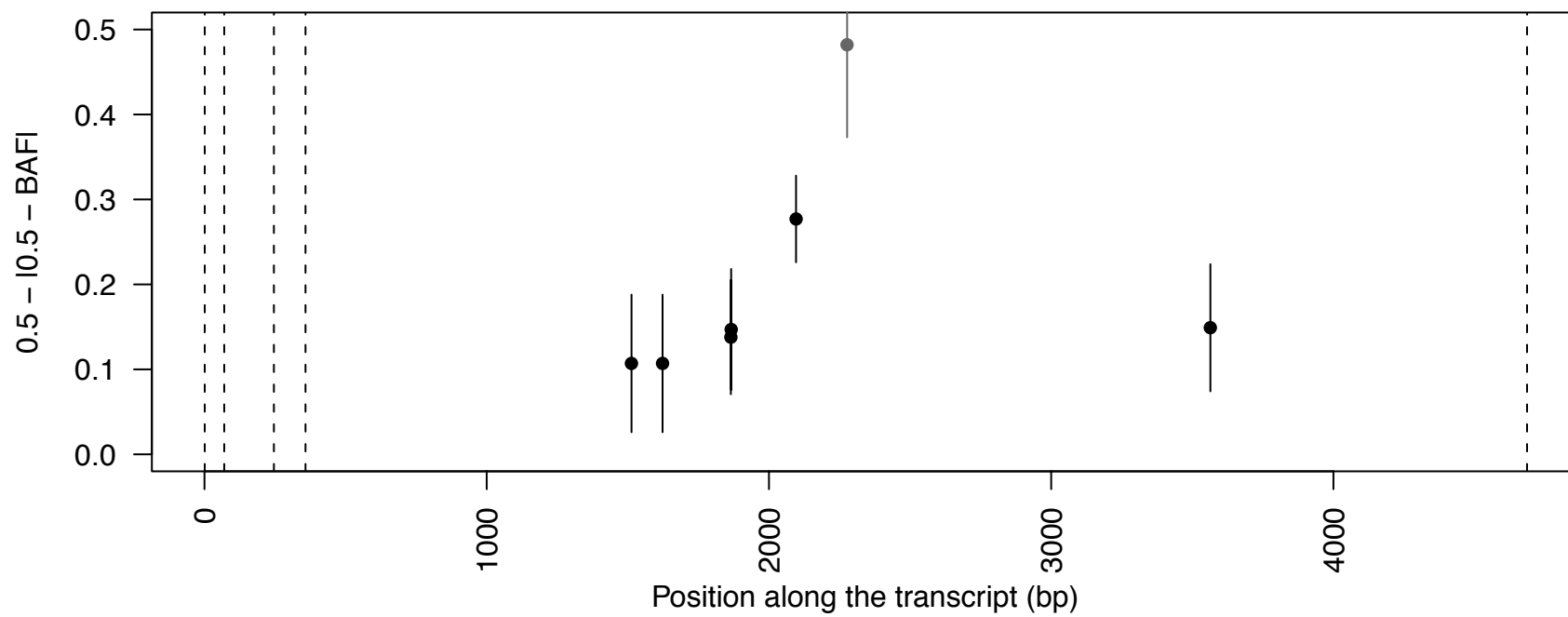

NR\_002819

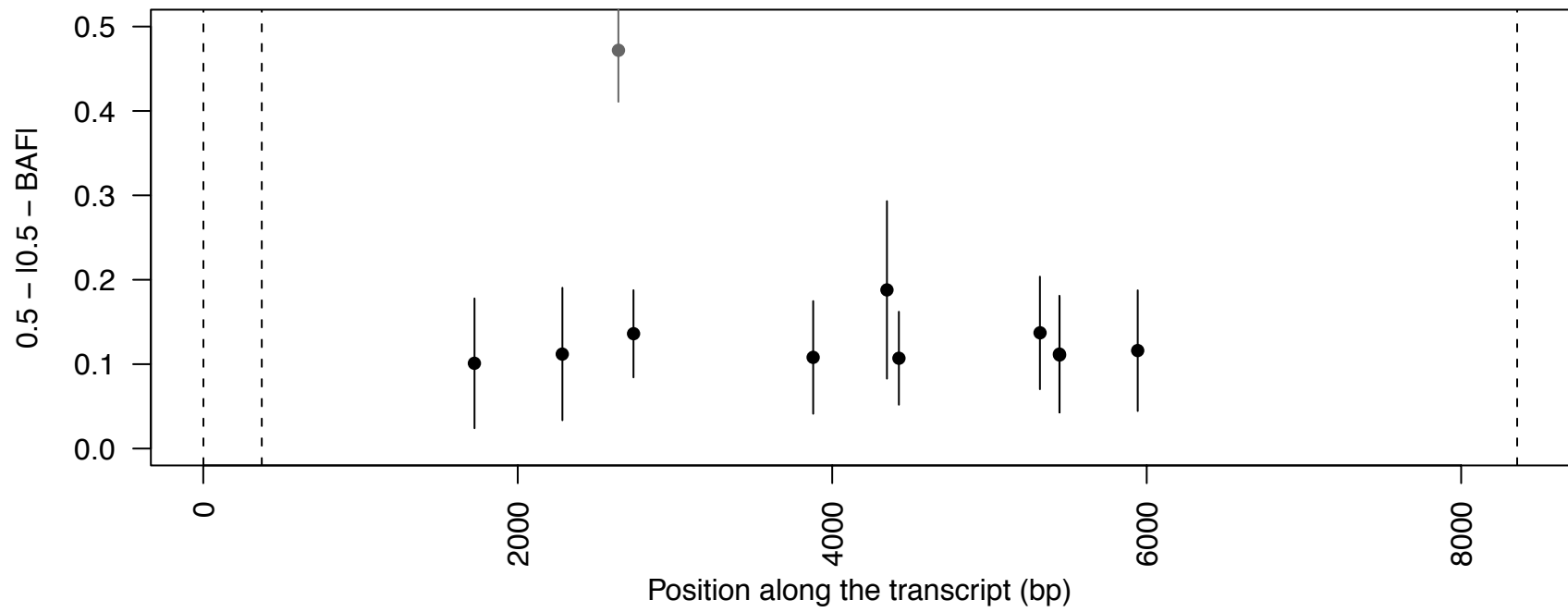

NR\_003255

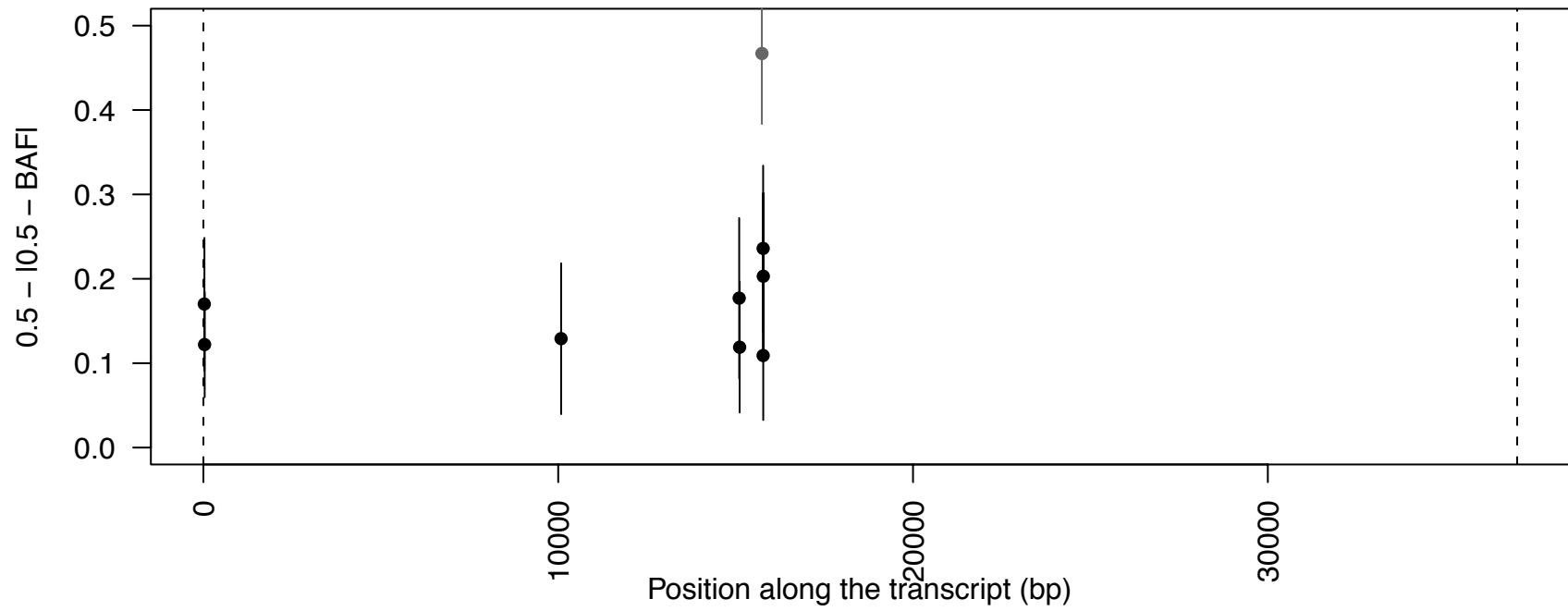

NR\_024425

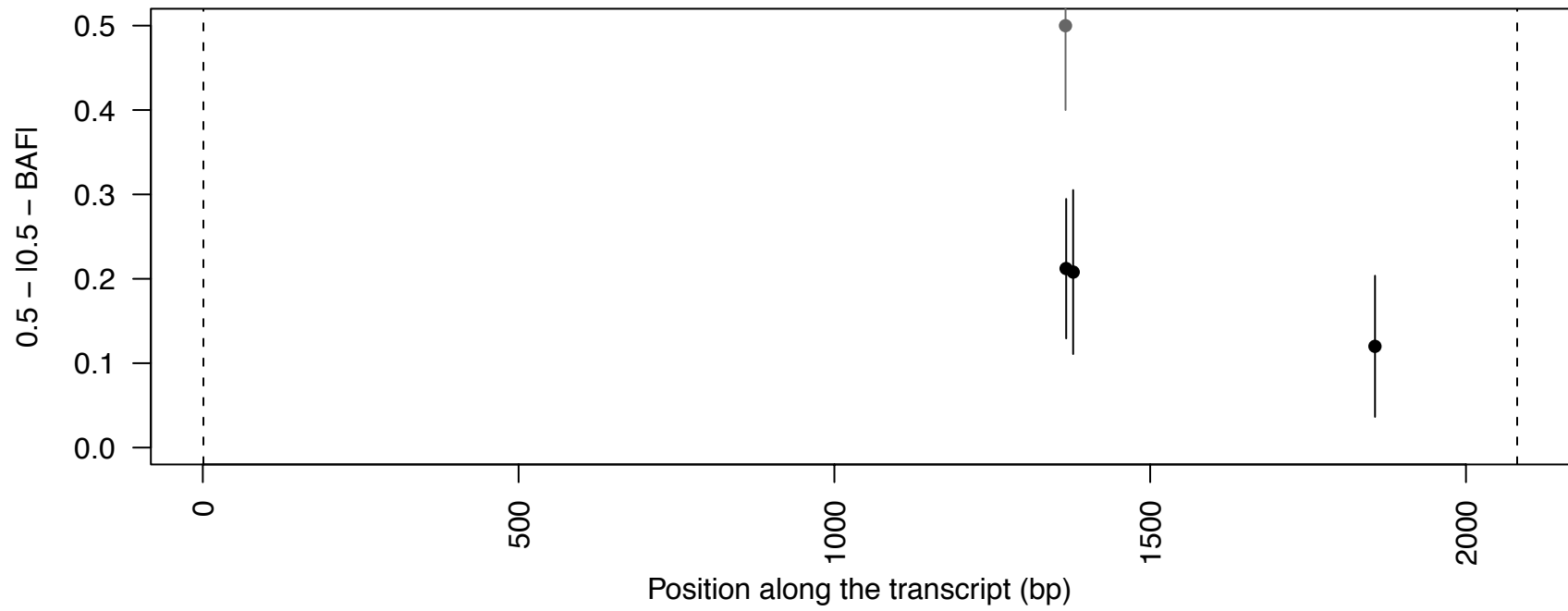

Supplement: Additional file 2 — Evidence of subclonal heterogeneity from RNA-Seq data across a collection of melanoma samples. Allelic imbalance along each gene demonstrates subclonal heterogeneity. The score of the non-reference nucleotide has been calculated as 0.5- | 0.5 - (#B/(#B+#A)) |, where #A and #B are the numbers of reads hosting the reference and non-reference nucleotide respectively. However, all the reported genes have a multimodal distribution of B-allele frequencies along at least one exon. The B-allele frequencies close to 0.5 have been marked in grey, B-alleles that significantly deviate from this cluster are considered acquired somatic mutations (black) and can be explained by subclonal heterogeneity. Vertical bars represent 95% confidence intervals. All non-reference nucleotides shown are supported by at least 100 reads. Vertical dashed lines mark the boundaries between exons in the transcript. [file 1471-2164-12-230-S2.PDF]
